# Differential somatic coding variant landscapes between laser microdissected luminal epithelial cells from canine mammary invasive ductal solid carcinoma and comedocarcinoma

**DOI:** 10.1186/s12885-024-13239-w

**Published:** 2024-12-18

**Authors:** Vivi Deckwirth, Sruthi Hundi, Marjo K. Hytönen, Sari Hannula, Pekka Ellonen, Pia Björkenheim, Antti Sukura, Hannes Lohi

**Affiliations:** 1 Present Address: Department of Veterinary Biosciences, Faculty of Veterinary Medicine, University of Helsinki, Helsinki, Finland; 2 Department of Medical and Clinical Genetics, Faculty of Medicine, University of Helsinki, Helsinki, Finland; 3 Present Address: Folkhälsan Research Center, Helsinki, Finland; 4 Institute for Molecular Medicine Finland FIMM, Helsinki, Finland; 5 Veterinary Teaching Hospital, Faculty of Veterinary Medicine, University of Helsinki, Helsinki, Finland

**Keywords:** Canine, Breast cancer, Single-cell sequencing, Cancer pathway, Laser microdissection, Comedocarcinoma, Solid carcinoma, Intratumor heterogeneity

## Abstract

**Background:**

Breast cancer (BC) is the most common cancer in women. Likewise, canine mammary tumors (CMT) represent the most common cancer in intact female dogs and develop in the majority spontaneously. Similarities exist in clinical presentation, histopathology, biomarkers, and treatment. However, CMT subtype-specific genomic background is less investigated. Here, we assess the genetic etiology of two histomorphological (HM) subtypes with BC counterparts, the CMT invasive ductal simple solid carcinoma (SC) and comedocarcinoma (CC), and compare the results with BC data.

**Methods:**

Groups of 11–13 transformed ductal luminal epithelial cells were laser-capture microdissected from snap-frozen invasive mammary SC and CC subtypes of one intact female dog. HM unaffected lobular luminal epithelial cells were controls. Single-cell whole genome libraries were generated using PicoPLEX and sequenced to compare the subtypes’ somatic coding variant landscapes with each other and with BC data available in COSMIC-CGC and KEGG. Furthermore, HM and immunohistochemical (IHC) subtype characteristics were compared with the genomic results.

**Results:**

The CC had six times more variants than the SC. The SC showed variants in adherens junction genes and genes of the MAPK, mTOR and NF-kappa-B signaling pathways. In the CC, the extracellular matrix (ECM) receptor interaction, cell adhesion, PI3K-Akt and cGMP-PKG pathways were enriched, reflecting the higher cellular malignancy. Affected pathways in both CMT subtypes overlapped with BC pathways in KEGG. Additionally, we identified *ATP6V1C2*, *GLYATL3, CARMIL3, GATAD2B, OBSCN, SIX2, CPEB3* and *ZNF521* as potential new subtype-distinct driver genes. Furthermore, our results revealed biomarker alterations in IHC in the basal/myoepithelial cell layer without respective genetic mutations, suggesting changes to their complex signaling pathways, disturbed regulative feedback loops or other silencing mechanisms.

**Conclusions:**

This study contributes to understanding the subtype-specific molecular mechanisms in the canine mammary invasive ductal simple SC and CC, and revealed subtype-specific molecular complexity for phenotypically similar characteristics. Several affected genes and signaling pathways overlapped with BC indicating the potential use of CMT as model for BC. Our findings emphasize the need for thorough characterization of cancer specimens with respect to translational cancer research, but also how insight into tumor heterogeneity will be crucial for the development of targeted prognostics and therapeutic interventions.

**Supplementary Information:**

The online version contains supplementary material available at 10.1186/s12885-024-13239-w.

## Background

Breast cancer (BC) is the most common cancer in women and surpassed lung cancer as the most fatal cancer in women in 2020 [1]. Research on BC has identified distinct molecular subtypes and therapeutically important biomarkers such as human epidermal growth factor receptor-2 (HER2), estrogen receptor-alpha (ERα) and progesterone receptor (PR) [2]. Sequencing technologies have revolutionized our understanding of the genetic and epigenetic etiology of BC with increasing amounts of data being cataloged in human cancer databases, such as the TCGA Portal and COSMIC [2–6]. Accumulation of variants across genomic coding and non-coding regions has provided insight into tumor progression, and has been important for the discovery of affected molecular driver pathways [7]. Furthermore, identifying BC subtype-specific somatic mutations has lead towards molecular targeted therapy and individual precision medicine [5, 8].

Canine mammary tumors (CMT) are the most common cancer in intact female dogs [9, 10]. Similarities exist in epidemiology, histopathology, molecular subtypes, pathogenesis, clinical presentation and therapy, suggesting that the dog is an important translational model for BC [11–19]. Recent CMT sequencing studies reported variants in genes such as *BRCA1, TP53*, *USH2A, FOXC2, cMYC* and in pathways such as PIK3-Akt, KRAS and HDR, as well as described molecular congruence of genes and pathways between BC and CMT [20–23]. Overlapping molecular etiologies are also described for other canine cancers, indicating that the tumor mutation burden is subtype-specific [24]. However, the suitability of the CMT model needs further exploration, including the somatic mutational landscapes of various tumor subtypes and their cell populations.

We targeted the CMT invasive ductal simple solid carcinoma (SC) and comedocarcinoma (CC) subtypes, because they have corresponding BC phenotypes [16, 25–27]. In both species, the malignant transformed ductal luminal epithelial cells proliferate to form either a solid sheet of pleomorphic cells filling the duct lumen (solid carcinoma) or display a peripheral solid sheet of pleomorphic cells with central necrosis (comedocarcinoma). In women, these phenotypes represent invasive ductal carcinomas mimicking ductal carcinoma in situ, i.e. with often unobvious invasion and metastasis in the histopathological evaluation [28–34]. Likewise, canine SC and CC associate with high vascular invasion and regional/distant metastasis rates at the time of diagnosis as well as shorter survival [16, 25, 26, 35–41]. We aimed to identify somatic variant landscapes from transformed ductal luminal epithelial cells from these two carcinoma subtypes, correlate with corresponding HM and IHC, and to compare the findings with recent discoveries in CMT and BC for possible shared etiology and differences.

## Materials and methods

### Canine clinical data

The samples originate from one intact, nulliparous 17-year-old mixed breed female dog. The right caudal 5th (inguinal) gland presented one mass of 20 × 30 mm in size at the pre-mastectomy clinical examination. The mass was noticed 2–3 weeks previously with following rapid growth and no previous history of mammary tumors. Therapeutic mastectomy comprised the 3rd-to-5th mammary glands *en-bloc* together with the inguinal fat pad. Additionally to the sampled neoplasia in the 5th mammary gland, the HM diagnosis revealed one simple tubular adenoma (ca. 3 mm in diameter) in the 3rd gland and one simple tubulo-papillary adenoma and one complex adenoma (both ca. 4 mm in diameter) in the 4th gland. Concurrent with the mastectomy, the dog was ovariohysterectomized. The right uterine horn displayed a well-delineated benign leiomyoma. According to the owner, the dog had no other illnesses or any medication.

### Sample collection

**Fig. 1:**
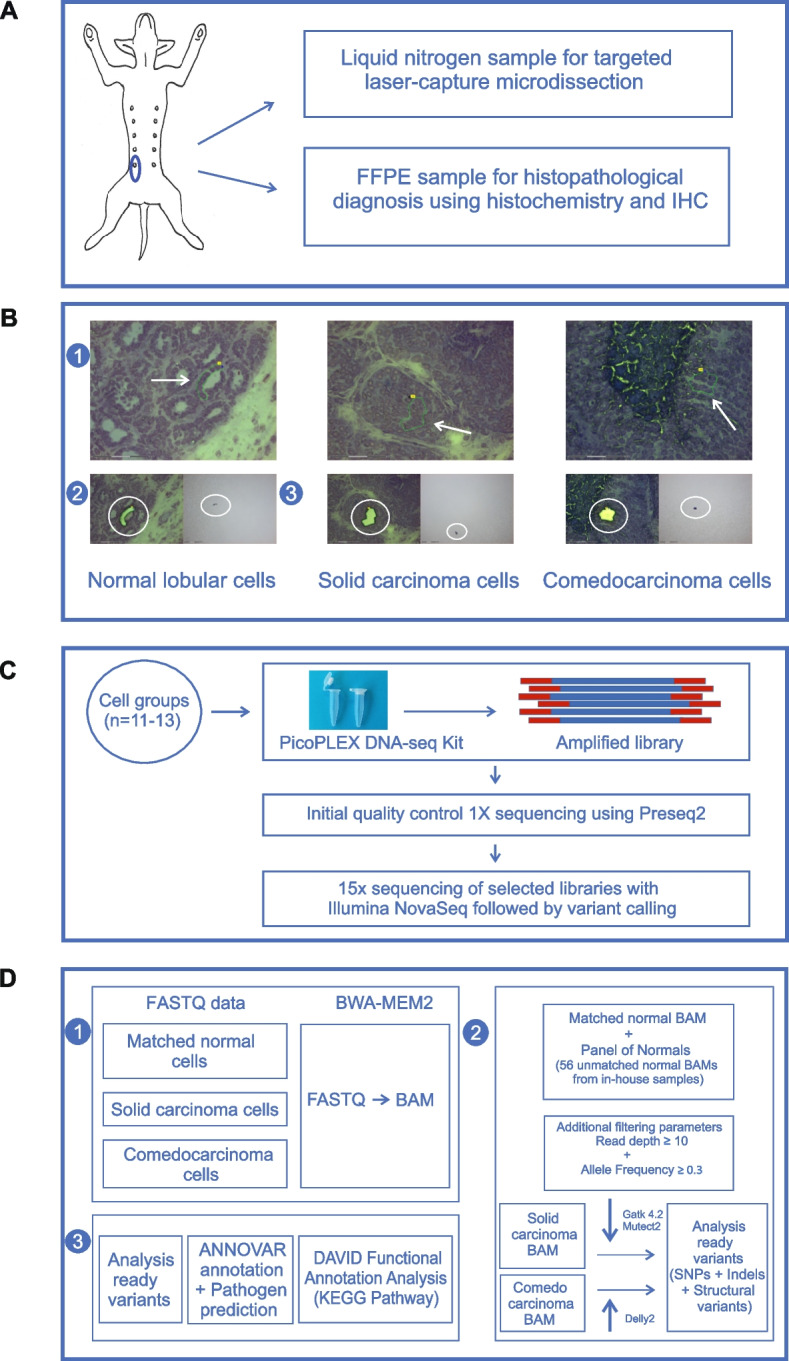
Flow diagram summarizing sample collection, whole genome sequencing, and data analysis. **A** Sample collection and tumor classification. Tumor sample from mastectomized right 5^th^ (inguinal) canine mammary gland for genomic analysis and tumor characterization using histochemistry and immunohistochemistry (IHC). FFPE: Formalin-fixed paraffin-embedded. **B** Laser-capture microdissection of cells for genomics. Targeted laser-capture microdissection (LCM) of luminal epithelial cell groups from normal lobular (control) and simple solid carcinoma as well as comedocarcinoma subtypes with documentation in three consecutive steps: step 1: Cellular capture area freehand marked with green. Scale bar 75 μm; step 2: Area after LCM. Scale bar 75 μm; step 3: Cap check of collecting tube for Cresyl-violet-stained sample material. Scale bar 150 μm. **C** Library construction and sequencing. DNA library construction, using the PicoPlex toolkit, from collected normal and carcinomatous cell groups. Analysis of libraries with Preseq2 and selection of further deep sequencing. WGS of selected libraries with Illumina Novaseq. **D** Discovery of somatic variants, genes and pathways. 1: FASTQ data aligned to the reference genome with BWA-MEM2 to generate BAM files. 2: Bioinformatics pipeline to identify somatic variants from invasive ductal solid and comedocarcinoma samples, including SNPs and indels with GATK Mutect2 and structural variants with Delly2, using a panel of normals comprising the matched normal lobular sample and 56 in-house samples. 3: Annotation of filtered variants to identify non-silent variants and further pathway analysis from KEGG using the DAVID online tool

Directly following therapeutic mastectomy, fresh tissue samples (ca. 5 × 10 mm in size) from macroscopically and palpable unaffected normal mammary gland and the 5th mammary gland neoplasia were collected for genetic analysis, snap-frozen in liquid nitrogen and stored at −80 °C (Fig. 1A). The remainder mastectomy tissue specimen together with two lymph nodes from the inguinal fat pad was submerged into 10% neutral-buffered formalin for 24 h at + 4 °C. The tissues were sampled and processed by automated (Tissue-Tek VIP Jr., Sakura) paraffin embedding (FFPE) and storage at RT (Fig. 1A) [42]. Sections adjacent to the samples for genetic analysis were specified for further investigation.

### Histopathology and pathologic staging

Canine and human classification criteria were used for histopathologic evaluation of mammary gland and lymph node samples from hematoxylin and eosin (HE) tissue sections of 4 μm [25, 27]. For this the slides were deparaffinized and rehydrated in xylen and alcohol series, followed by a rinse for 2 min in distilled aqua prior staining for 10 min in Meyer’s Hematoxylin (Cat. 180210 Reagena) and subsequent bluing step under running tap-water for 10 min. After another rinsing step in distilled aqua, eosin (Eosin Y, C.I. 45380, Cat. 1.15935, Merck) counterstain was applied for 2 min, followed by dehydration in alcohol series, clearing in xylene and mounting with Pertex (Cat. 00811, Histolab). The procedure was automated (Leica AutoStainer XL, Leica Biosystems). The neoplasia focality, extent, type of invasion and resection margins were assessed, and the histologic grade and pathologic stage determined using semiquantitative tripartite scoring methods as described previously [15, 25, 42–44]. The evaluation was performed by VD. Shortly, for the histologic grade were considered glandular (acinar) and tubular differentiation, nuclear pleomorphism and mitotic count. For the glandular/tubular differentiation, the percentage of area with structures showing central lumen surrounded by polarized cells within the tumor was evaluated. Cut-off points were at 75% for discriminating between majority of tumor area and moderate differentiation, and at 10% for little or no glandular/tubular formation. For the nuclear pleomorphism, the size and uniformity of the neoplastic cells were assessed at a location displaying highest degree of pleomorphism compared to adjacent normal tissue. For the mitotic count, only clear mitotic figures were counted from 10 high-power fields (40 × objective; field diameter 0.57 mm). For the pathologic stage (pTNM), the extent of the primary tumor (categories: not possible to assess / no primary tumor / in situ carcinoma / tumor size in greatest dimension / tumor with direct extension to chest wall or skin), regional lymph node status (categories: not possible to assess / no regional lymph node metastasis / regional micrometastases or metastases) and distant metastasis (categories: not present / present) were determined. Possible lymph node affection by macrometastases (> 0.2 cm), micrometastases (> 0.2 mm to 0.2 cm and/or > 200 cells), or isolated epithelial tumor cells (ITC; ≤ 0.2 mm and ≤ 200 cells) was evaluated using pan-cytokeratine (Clone AE1/AE3) and high-molecular-weight cytokeratine (Clone 34βE12) IHC [43]. Further classification based on Masson’s trichrome stain and IHC biomarkers including hormone receptors (e.g. ERα, PR, HER2), luminal epithelial (e.g. CK8, CK18, CK19, E-Cadherin) and basal/myoepithelial markers (e.g. CK5, p63, Calponin, α-SMA) as well as the proliferation indicator Ki-67. Neuroendocrine differentiation was excluded using Chromogranin A and Synaptophysin IHC [45–48].

### Immunohistochemistry

FFPE serial Sects. (4 µm) were prepared on Menzel Superfrost Plus Adhesion microscope slides (Cat. J1800AMNZ, Thermo Fisher Scientific) and baked at + 37 °C o/n, deparaffinized and rehydrated in xylen and alcohol series (Leica AutoStainer XL, Leica Biosystems) before antigen retrieval. Used primary antibodies and conditions are detailed in Table S1. After cooling to RT, the slides were blocked for 10 min with 3% hydrogen peroxide in PBS (Cat. BP339-4, Fisher Scientific) and rinsed twice with TBS (Cat. J640-4L, VWR Chemicals) + Tween (Cat. P1379, Sigma). The BrightVision Poly-HRP-Anti Ms/Rb/Rt IgG Kit (Cat. DPVO110HRP, Immunologic) was used for staining at RT, and 3.3´-diaminobenzidine-tetrahydrochloride (Cat. VWRKBS04-110, Immunologic) was applied for 5 min for visualization. Harris hematoxylin (Cat. HX57998853, Merck) counterstain was used for 10 s, followed by dehydration, and mounting with Pertex (Cat. 00811, Histolab). Positive controls for ChromograninA and Synaptophysin were canine pancreatic tissue. For all other antibodies, adjacent canine normal mammary was an internal positive control. For negative controls antibody diluent only was used.

Evaluation of ERα, PR, HER2 and Ki-67 followed human and canine guidelines, with consideration of HER2 scores 2 + and 3 + as positive [9, 49–53]. Thresholds for ERα and PR positivity were ≥ 1% of neoplastic cells displaying nuclear staining and for HER2 positivity > 10% of neoplastic cells showing complete membrane staining. Other biomarkers were evaluated negative or positive according to absence or presence of staining, and the positive reaction was further categorized as high, moderate, or weak.

### Cell isolation using laser-capture microdissection

Samples for genetic analysis were stored at −80 °C until LCM. Prior to cryosectioning, Polyethylene Naphthalate 1.0 (PEN)-MembraneSlides (Cat. 415190–9041-000, Zeiss, Göttingen, Germany) were UV-radiated for 30 min, and tissue samples were equilibrated to cryosection temperature (−27 °C) and embedded into Optimal Cutting Temperature Compound (Cat. 361603E, VWR International, Radnor, USA). Sections of 5 μm were cryosectioned (CM1900 Cryostat, Leica, Nussloch, Germany) onto the PEN-MembraneSlides followed by cresyl violet staining. For this, the slides were immersed in 70% ethanol, followed by a dip for 10 s into 1% cresyl violet acetate solution (Cat. 1 A 396, Chroma-Gesellschaft Schmid & Co., Stuttgart-Untertürkheim, Germany). The slides were dehydrated with dipping into 70% ethanol and 100% ethanol and dried completely before LCM. Separate solutions were prepared for normal and pathologic samples.

The cresyl violet stain allowed reliable histomorphologic identification of the respective sample features and manual selection of the cell populations for genomic analysis under visual control using a 40 × objective (Zeiss PALM MicroBeam instrument, Zeiss, Munich, Germany) (Fig. 1B, Table 1). The laser-pressure catapulted microdissected cell populations were dry-collected into sterile PALM opaque AdhesiveCap microfuge tubes (Cat. 415190- 9201–000, Zeiss, Göttingen, Germany) for immediate next generation sequencing (NGS) library construction. Stepwise microphotographs and data of collected cells were documented (Fig. 1B, Table 1).

**Table 1:** Laser-capture microdissection (LCM) sample data (PALMRobo element list, Version V 4.5.0.9)

Sample ID	Type	Number of cells	LCM area (μm2)	Histopathological diagnosis
79	Freehand	13	506	Lobular normal luminal epithelium
87	Freehand	12	532	Lobular normal luminal epithelium
142	Freehand	13	900	Ductal solid carcinoma, transformed intraluminal epithelium
194	Freehand	11	793	Ductal comedocarcinoma, transformed intraluminal epithelium

Sample ID refers to the sample number indicated in stepwise microphotographs shown in Fig. 1B. Type – Freehand stands for freehand marking of LCM sample on tissue using a 40 × objective

### DNA library construction

The LCM cell material was immediately processed into NGS libraries using the PicoPLEX DNA-seq Kit (Cat. R300381, Rubicon Genomics, Ann Arbor, MI USA) according to the manufacturer’s protocol (Fig. 1C). This was executed by the Institute for Molecular Medicine Finland (FIMM, Helsinki, Finland). Libraries chosen for whole genome sequencing are listed in Table 2.

**Table 2:** PicoPLEX (Rubicon Genomics, Ann Arbor, MI USA) genomic libraries chosen for deeper whole genome sequencing and their respective NCBI Sequence Read Archive (SRA) identifications (ID)

LCM ID	PicoPLEX library ID	NCBI SRA ID	Histopathological diagnosis
79	DOG_MAMMA_28	SRR22765702	Lobular normal luminal epithelium
87	DOG_MAMMA_32	SRR22765701	Lobular normal luminal epithelium
142	DOG_MAMMA_1	SRR22765700	Ductal solid carcinoma, transformed intraluminal epithelium
194	DOG_MAMMA_10	SRR22765699	Ductal comedocarcinoma, transformed intraluminal epithelium

PicoPLEX library ID refers to the sample numbers in Additional File 2. LCM stands for laser-capture microdissection

### Whole genome sequencing

NGS libraries were initially sequenced at low coverage (~ 1x) to test for yield and utility for further sequencing. The acquired FASTQ files were aligned using BWA-MEM2 (version 2.2.1), and the resulting BAM files were evaluated for WGS using Preseq2 lc_extrap (version 2.0.0) [54] (Fig. S1). Two normal mammary lobular luminal epithelial libraries and one library each for the transformed luminal epithelium from ductal simple SC and CC subtypes were selected for deeper sequencing (Table 2). The normal lobular libraries were sequenced at 15X and 12X coverages, and the SC and CC at 9X and 15X coverages, respectively. Sequencing was performed using Illumina Novaseq S1 PE151, and FASTQ files were produced using the bcl2fastq tool (version 2.20). Sequencing was executed by the Institute for Molecular Medicine Finland (FIMM, Helsinki, Finland). See Figs. 1C and D for a flowchart of the bioinformatic approach**.**

### Somatic variant calling

The produced library FASTQ files were aligned with GSD1.0 (Accession ID: GCA_011100885.1) [55] as the base reference and supplemented with Y chromosome from ROS_Cfam1.0 (Accession ID: GCA_014441545.1) with BWA-MEM2 to make BAM. Best practices guideline from GATK4.2 Mutect2 were followed to identify somatic variants from the BAM files of SC and CC with a false discovery rate 0.1 using the BAM from unaffected normal mammary lobule samples as a matched normal since they originated from the same dog [56]. A panel of normal (PON) samples was used to capture technical artifacts and improve variant calling analysis results. To build the PON, Mutect2 was used to call somatic variants from 56 in-house WGS (Illumina) blood sample data. This was followed by LearnReadOrientationModel and FilterMutectCalls from GATK to filter variants due to sequencing artifacts. Variants with an allelic depth greater than or equal to 0.3 and a minimum read depth of 10 were selected to exclude the false positive risk due to sequencing errors. An in-house germline database was provided as a germline resource for Mutect2. The FASTQ data used to create the germline database has been made available in the NCBI SRA BioProject PRJNA913118.

Structural variants (SVs) were called using a somatic structural variant pipeline from DELLY2 (Version 1.1.6) [57] and included insertions, deletions, duplications and intrachromosomal rearrangements. A PON was created to filter false positives and germline structural variants efficiently using BAM files from normal mammary lobules and 56 in-house samples. The FASTQ data used to create the PON has been made available in the NCBI SRA BioProject PRJNA913118. Imprecise SVs were filtered out according to the DELLY pipeline. A precise set of SVs was selected after validation using the IGV [58] and Ribbon genome browsers [59]. The final set of precise variants that included single nucleotide polymorphisms (SNPs), indels and SVs were annotated with gene information from RefSeq databases using ANNOVAR (Version: $Date: 2020–06–07) [60] with annotation database built from GSD1.0 (Accession ID: GCA_011100885.1) as the base reference and supplemented with Y chromosome from ROS_Cfam1.0 (Accession ID: GCA_014441545.1). See Figs. 1C–D for the bioinformatic approach.

### Variant pathogenicity prediction

Missense variants predicted to be possibly damaging (less confident prediction) variants and probably damaging (more confident prediction) variants by PolyPhen-2 [61, 62] and other loss-of-function variants, such as frameshift, non-frameshift, start- / stop-loss and stop-gain variants by ANNOVAR (Version: $Date: 2020–06–07) [60] annotations were considered as pathogenic variant set.

### Pathway enrichment analysis

The genes with non-silent variants were subjected to functional analysis using DAVID v6.8 (https://david.ncifcrf.gov/), with the selected identifier as OFFICIAL_GENE_SYMBOL, species as Canis lupus familiaris and list type as ‘Gene List’. Functional annotation summary results for KEGG_PATHWAY with EASE score /p-value set to 0.05 were downloaded. The p-value of the pathways represent the raw significance level of pathways being overrepresented in this dataset providing initial insights into potential enriched pathways.

### Driver gene prediction

The non-silent variant set was further analyzed with OncodriveCLUSTL (version 1.1.4) to identify cancer drivers [7]. The tool was adapted for the canine genome with the above-mentioned reference and ran with parameters set to k-mer = 3, smooth_window = 11, cluster_window = 11, n_simulations = 1000, simulation_mode = mutation_centered and simulation_window = 31 to identify driver genes.

### Determination of ERBB2 amplification status

VarScan2 (version 2.4.2) was employed to identify amplification and deletion of *ERBB2* in SC and CC with two matched normal samples as control [63].

### Comparison with known literature and databases

Variant gene lists were compared with CMT and BC literature retrieved from the PubMed -database (https://pubmed.ncbi.nlm.nih.gov/) based on gene specific search and used for the discussion. To determine the number of genes from our precise variant list that have been previously reported in BC and CMT, we compared our variant gene list with the COSMIC Cancer Gene Census (CGC) and the KEGG (Kyoto Encyclopedia of Genes and Genomes) CMT pathway (cfa05224). Human gene orthologs were retrieved with Ensembl BioMart [64] for comparison with COSMIC-CGC [65] gene list, including Tier 1 and Tier 2 categories.

## Results

### Canine tumor classification as invasive ductal simple solid carcinoma and comedocarcinoma

**Fig. 2:**
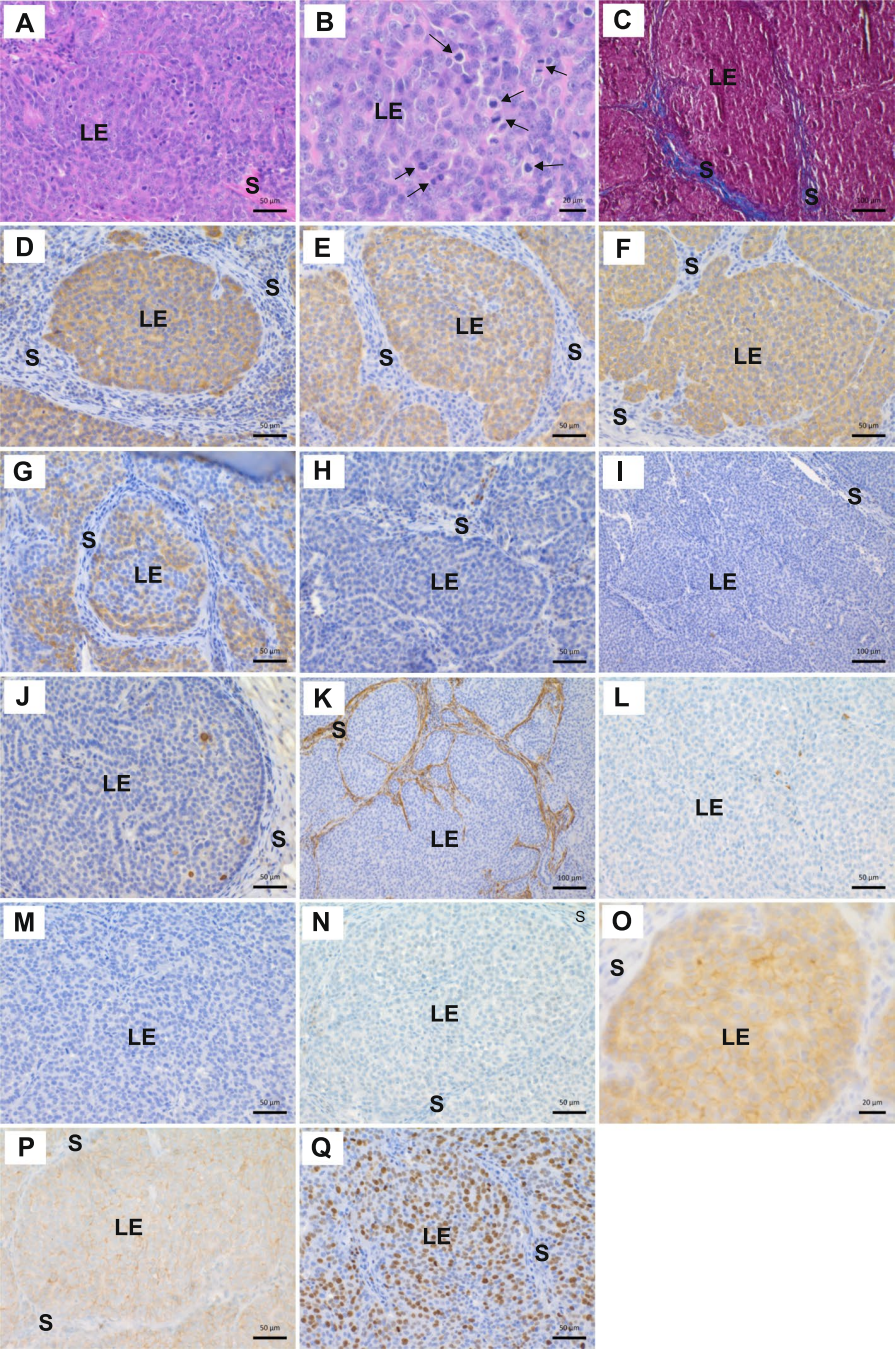
Histopathology and biomarker immunohistochemistry of canine invasive ductal solid carcinoma phenotype. Structures have been annotated as follows: LE=luminal epithelium, S=stroma. **A** Solid carcinoma with scant supporting stroma. HE. Scale bar 50 μm. **B** High-grade morphology and high mitotic activity (3–11/HPF 40x objective). Some mitoses are marked by arrows. HE. Scale bar 20 μm. **C** Solid growth pattern with scant intervening stroma (blue). Masson’s trichrome stain. Scale bar 100 μm. **D** Moderate epithelial E-Cadherin expression. Scale bar 50 μm. Counterstain Harris hematoxylin. **E** Moderate luminal epithelial CK8 expression. Scale bar 50 μm. Counterstain Harris hematoxylin. **F** Moderate luminal epithelial CK19 expression. Scale bar 50 μm. Counterstain Harris hematoxylin. **G** Partial weak-to-moderate luminal epithelial CK18 expression. Scale bar 50 μm. Counterstain Harris hematoxylin. **H** Peripheral loss of basal / myoepithelial nuclear p63 expression. Up right residual entrapped normal ductal nuclear expression. Scale bar 50 μm. Counterstain Harris hematoxylin. **I** Loss of basal / myoepithelial CK5 expression. Single positive entrapped cells are present. Scale bar 100 μm. Counterstain Harris hematoxylin. **J** Loss of basal / myoepithelial CK14 expression. Single entrapped CK14-positive cells are present. Scale bar 50 μm. Counterstain Harris hematoxylin. **K** Loss of basal / myoepithelial and increased stromal myofibroblastic α-SMA expression. Scale bar 100 μm. Counterstain Harris hematoxylin. **L** Loss of basal / myoepithelial Calponin expression. Entrapped single positive cells are present. Scale bar 50 μm. Counterstain Harris hematoxylin. **M** Negative ERα expression. Scale bar 50 μm. Counterstain Harris hematoxylin. **N** Negative PR expression. Scale bar 50 μm. Counterstain Harris hematoxylin. **O** Weak-to-moderate complete membrane HER2 expression in >10 % of tumor cells. Score 2+. Scale bar 20 μm. Counterstain Harris hematoxylin. **P** Weak-to-moderate complete membrane EGFR expression in >10 % of tumor cells. Scale bar 50 μm. Counterstain Harris hematoxylin. **Q** High score nuclear Ki-67 expression. Scale bar 50 μm. Counterstain Harris hematoxylin

**Fig. 3:**
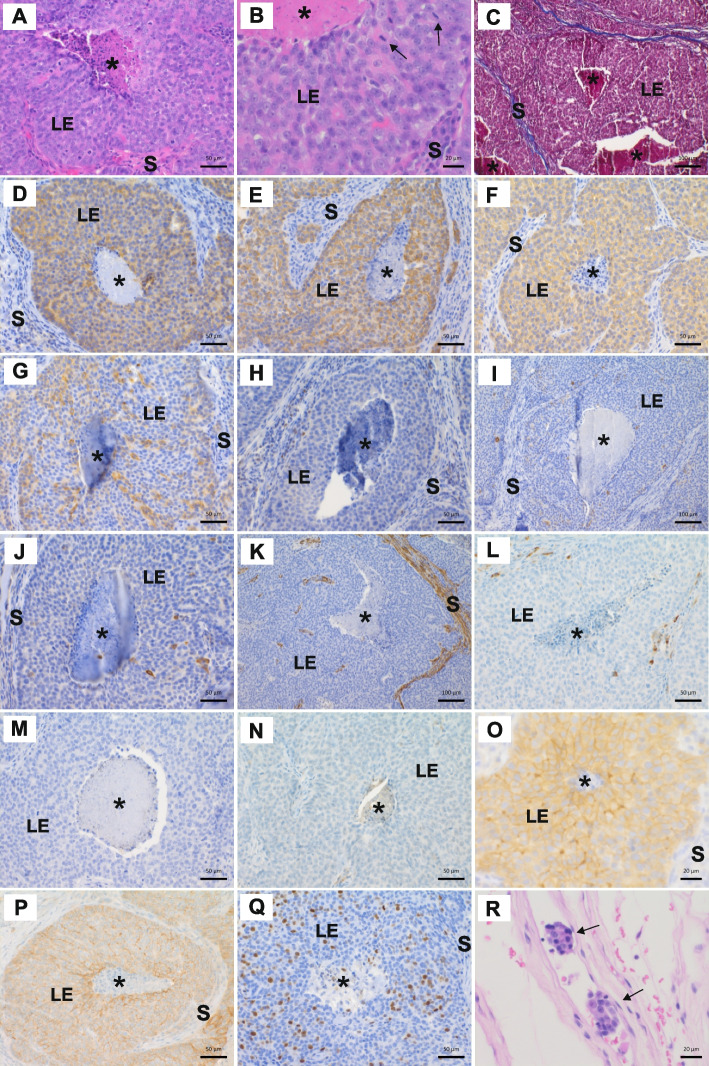
Histopathology and biomarker immunohistochemistry of canine invasive ductal comedocarcinoma phenotype. Structures have been annotated as follows: LE=luminal epithelium, S=stroma, asterix=necrosis. **A** Comedocarcinoma with central necrosis and scant supporting stroma. HE. Scale bar 50 μm. **B** High-grade morphology and intermediate mitotic activity (2–6/HPF 40x objective). Some mitoses are marked by arrows. HE. Scale bar 20 μm. **C** Solid growth pattern with central necrosis and scant intervening stroma (blue). Masson’s trichrome stain. Scale bar 100 μm. **D** Moderate epithelial ECadherin expression. Scale bar 50 μm. Counterstain Harris hematoxylin. **E** Moderate luminal epithelial CK8 expression. Scale bar 50 μm. Counterstain Harris hematoxylin. **F** Moderate luminal epithelial CK19 expression. Scale bar 50 μm. Counterstain Harris hematoxylin. **G** Partial weak luminal epithelial CK18 expression. Scale bar 50 μm. Counterstain Harris hematoxylin. **H** Peripheral loss of basal / myoepithelial nuclear p63 expression. Single positive cells are present. Scale bar 50 μm. Counterstain Harris hematoxylin. **I** Loss of basal / myoepithelial CK5 expression. Single entrapped CK5-positive cells are present. Scale bar 100 μm. Counterstain Harris hematoxylin. **J** Loss of basal / myoepithelial CK14 expression. Single entrapped CK14-positive cells are present. Scale bar 50 μm. Counterstain Harris hematoxylin. **K** Loss of basal / myoepithelial and increased stromal myofibroblastic α-SMA expression. Scale bar 100 μm. Counterstain Harris hematoxylin. **L** Loss of basal / myoepithelial Calponin expression. Single positive cells are present, partially entrapped. Scale bar 50 μm. Counterstain Harris hematoxylin. **M** Negative ERα expression. Scale bar 50 μm. Counterstain Harris hematoxylin. **N** Negative PR expression. Scale bar 50 μm. Counterstain Harris hematoxylin. **O** Weak-to-moderate complete membrane HER2 expression in >10 % of tumor cells. Score 2+. Scale bar 20 μm. Counterstain Harris hematoxylin. **P** Weak-to-moderate complete membrane EGFR expression in >10 % of tumor cells. Scale bar 50 μm. Counterstain Harris hematoxylin. **Q** Intermediate score nuclear Ki-67 expression. Scale bar 50 μm. Counterstain Harris hematoxylin. **R** Peritumoral neoplastic lymphovascular embolization. Tumor cell emboli are marked by arrows. HE. Scale bar 20 μm

The tumor comprised two HM subtype components determined as solid carcinoma and comedocarcinoma based on HE and Masson’s trichrome (Figs. 2A-C, 3A-C). Transformed epithelial cells were confined within the luminal area with occasional focal discontinuity and invasion (Figs. 2A and 3A). Expanded ducts showed cells in solid growth pattern and multifocal solid cell areas with central necrosis. Thin stromal support localized in-between (Figs. 2A, C, 3A, C). Overlapping transformed cells displayed high pleomorphy with increased size and large pleomorphic nuclei, peripheral cytoplasm and increased number of nucleoli (Figs. 2B, 3B). Mitotic figures were increased and showed aberrancies (Figs. 2B and 3B). Apoptosis was more frequent in the CC. For both subtypes, the nuclear grade was high, and the cellular grade poorly differentiated (Figs. 2B, 3B). Multifocal peripheral moderate chronic inflammatory infiltrate was observed (Data not shown). Lymphovascular embolization was present (Fig. 3R). Inguinal lymph nodes were negative for isolated tumor cells, micro- and macrometastases (Data not shown). Pre-surgical clinical examination included no thorax radiographs for metastasis evaluation. The pathologic stage of the neoplastic disease was determined as T2N0M- according to the canine and human systems [15, 44].

E-Cadherin positivity confirmed ductal origin for the transformed cells in both subtypes [66]. The staining intensity was peripheral strong and centrally weaker (Figs. 2D, 3D). In both subtypes, the transformed cells were cytokeratin (CK) 8 and 19 positive and multifocal positive for CK18, but negative or with single entrapped positive cells for CK5 and CK14, indicating luminal epithelial origin of the transformed cell populations as compared to basal epithelial cell origin (Figs. 2E–G, I–J, 3E–G, I–J) [67]. In the peripheral basal cellular compartment both subtypes presented complete or almost complete loss of the basal/myoepithelial markers CK5, CK14, p63 and Calponin (Figs. 2H–J, L, 3H–J, L). Furthermore, suggestive of myoepithelial loss, α-SMA expression was decreased or lost basally. However, the biomarker was increased in the stroma, indicative of myofibroblastic reaction (Figs. 2K, 3K). Masson’s trichrome showed only suggestive rudimentary basement membrane (BM) (Figs. 2C, 3C). Invasion was determined as the absence of myoepithelial cells and/or extension through the BM [25, 27, 68].

IHC for PR and ERα were negative and HER2 was determined as equivocal (2 +) positive (Figs. 2M–O, 3M–O) [9, 50–52]. Unfortunately, we could not assess possible *ERBB2* amplification using in situ hybridization according to BC diagnostic recommendations [51, 52]. However, the implementation of VarScan 2 did not detect any amplification or deletion across the subtypes (Table S2). Manual inspection of *ERBB2* in IGV using BAM files showed some coverage difference between control and tumor samples but did not reveal any strong signs of duplication (Figure S2). EGFR was membranous and weak-to-moderate in both subtypes (Figs. 2P, 3P). Negativity for Chromogranin A and Synaptophysin excluded carcinoma subtypes with neuroendocrine differentiation as described for both species (Data not shown) [45–48]. Expression of Ki-67 showed higher proliferative activity in transformed cells of both subtypes compared with normal tissue (Figs. 2Q, 3Q). Interestingly, the percentage of Ki-67-positive tumor cells was higher in SC (49%, high) than CC (13%, intermediate) as determined using the global scoring method [49, 51, 53]. Normal mammary IHC biomarker expression patterns are shown in Figure S3.

According to the canine classification system, the tumor consisted of invasive ductal simple carcinoma of the comedo subtype with areas of solid subtype [25]. According to human breast tumor classification criteria, this corresponds to the subtype of invasive carcinoma of no special type (ductal carcinoma NST) comprising a mixed phenotype with comedo and solid components [48]. Considering molecular subtypes, the tumor was negative for hormone receptors ERα and PR, but equivocal positive (2 +) for HER2 and positive for luminal epithelial biomarkers CK8, CK18 and CK19 [13, 69].

### Differential somatic variant landscapes between invasive ductal solid and comedo subtypes

**Fig. 4:**
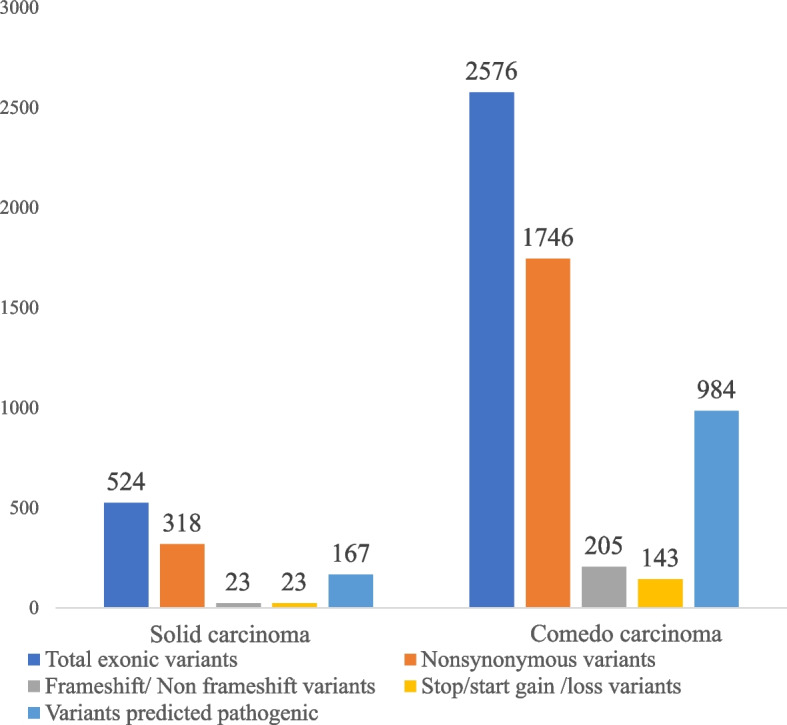
Somatic coding variants in canine invasive ductal solid and comedocarcinoma. Total exonic variants includes non-silent and silent variants. Amounts of the non-silent variant categories of non-synonymous, frameshift / non-frameshift and stop / start-gain / -loss variants and numbers of predicted pathogenic variants are presented

Altogether, 227,962 somatic variants were identified in the WGS of transformed luminal epithelial cells from SC and CC when filtered against the matched non-tumorigenic genome. Combining both subtypes, we found 3100 somatic coding variants, including SNVs and indels (Fig. 4). The SC carried 524 exonic variants of which 364 were non-silent variants (Fig. 4). Among the 364 non-silent variants were 318 (87.36%) non-synonymous (NSV), 12 (3.30%) frameshift deletion, four (1.10%) frameshift insertion, four (1.10%) non-frameshift insertion, two (0.55%) non-frameshift deletion, one (0.27%) non-frameshift substitution, 19 (5.22%) stop-gain, three (0.82%) stop-loss and one start-loss (0.27%) variants. The CC had 2576 exonic variants, including 2094 non-silent variants, about 6 times more than the solid subtype. Of the 2094 non-silent variants in CC, 1746 (85.42%) were NSV, 189 (9.03%) frameshift deletion, four (0.19%) frameshift insertion, four (0.19%) non-frameshift deletion, one (0.05%) non-frameshift insertion, seven (0.33%) non-frameshift substitution, 133 (6.35%) stop-gain, nine (0.43%) stop-loss and one (0.05%) start-loss variant (Fig. 4).

According to the PolyPhen-2 prediction analysis of the 318 NSVs in SC, 197 were predicted as benign, 78 as possibly damaging and 43 as probably damaging. Of the 1746 NSVs in CC, 1110 were predicted as benign, 412 as possibly damaging and 224 as probably damaging. Thus, the SC had 31.87% (167 out of 524) of exonic variants predicted as pathogenic (probably damaging, possibly damaging, frameshift and non-frameshift variants, stop-loss, stop-gain and start-loss), and the CC had 38.19% (984 out of 2576) of exonic variants predicted as pathogenic (Tables S3A, S3B). We also found 196 SVs, of which 36 belonged to SC and 160 to CC. Of these, four inversions and one duplication localized within coding regions in the SC, and two duplications, two deletions and four inversions in the CC (Tables S4A, S4B).

### Differentially affected pathways between invasive ductal solid and comedo subtypes

**Fig. 5:**
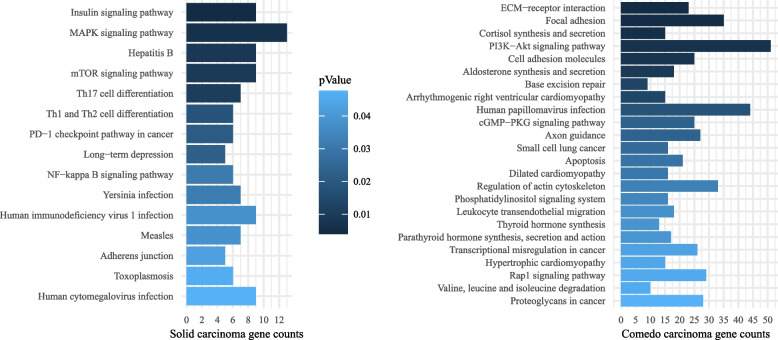
KEGG pathway analysis identified distinct enriched pathways for canine invasive ductal solid and comedocarcinoma. Color shade intensity increases with p-value significance. More significant pathways localize to the top. Note different scales in the left and right panels

Non-silent coding variants affected 344 genes in the SC and 1863 genes in the CC, with more than one variant identified in 17 genes of the SC and in 202 genes of the CC (Tables S3A and S3B). We found 73 genes with non-silent variants in both subtypes (Table S5). We conducted gene enrichment analysis to find subtype-specific affected pathways. The KEGG pathways enriched (*p* < 0.05) gene lists showed 15 enriched pathways (gene count ≥ 5) for SC and 24 enriched pathways (gene count ≥ 5) for CC as shown in Fig. 5 and listed in Tables S6A and S6B. These results indicate that more genes and pathways are affected in the CC than in the SC, with only a minor number of mutual genes involved.

### Shared and unique predicted driver genes between invasive ductal solid and comedo subtypes

Using OncodriveCLUSTL, we found shared and unique driver genes in the two subtypes (Table S7). *NEO1* was identified as a driver in both subtypes, *ATP6V1C2* as a driver in SC and *GLYATL3*, *CARMIL3*, *GATAD2B*, *OBSCN*, *SIX2*, *CPEB3* and *ZNF521* as drivers in CC. These results show more driver genes in the CC than in the SC.

### Data comparison to annotated human and canine databases

We mapped the gene sets against available databases to assess whether the affected genes and pathways were previously associated with BC and CMT. We found 23 genes overlapping with the CMT pathway (cfa05224) in the KEGG database [70]. Of these, two genes were shared by both canine subtypes; three were distinct for SC and 18 for CC. Next, we retrieved the human orthologs for our genes and compared them with the Tier 1 and Tier 2 lists of 736 genes in COSMIC-CGC [71]. About 7.27% (25 out of 344) of the SC genes and 6.39% (119 out of 1863) of the CC genes matched (Table 3). These results show that the few overlapping pathways and genes are mostly subtype specific (Table S8).

**Table 3:** The number of overlapping genes identified with CMT KEGG-cfa05224 and BC COSMIC-CGC databases

	**Solid carcinoma**	**Comedocarcinoma**	**Common in both subtypes**
COSMIC-CGC	25	119	8
KEGG-cfa05224	5	20	2
Total in this study	344	1863	73

Taken together, our results demonstrate that despite the observed close similarities in HM and IHC biomarker panel expression, the investigated CMT subtypes are molecularly distinct from each other, with genomic alterations more abundant and diverse in the CC than in the SC. Moreover, both subtypes’ gene and pathway affection overlap with BC.

## Discussion

CMTs are suggested as a translational model for BC research. However, the dog subtype specific genetic etiology is less investigated. Here, we compared the somatic variant landscape of two CMT subtype components of an invasive mixed phenotype tumor in groups of transformed ductal epithelial cells from a single dog with each other and human data. Furthermore, we performed histopathological classification and IHC to characterize both subtypes’ phenotypes and compared these with the genomic data.

Phenotypically, both subtypes displayed ductal origin (E-Cadherin^+^) with aberrant ductal size, morphology and cell components, suggesting dysfunction in ductal morphogenesis and epithelial cell lineage differentiation (Figs. 2D and 3D) [67]. The expanded transformed epithelial cell populations expressed the luminal epithelial biomarkers CK8^+^, CK18^±^ and CK19^+^ with a hormone receptor status of ERα^−^, PR^−^, HER2^+^ and EGFR^+^ (Figs. 2E-G, M-P, 3E-G, M-P). We observed no alterations to *ESR1* encoding for ERα or *PGR* encoding for PR in the investigated subtypes, inferring some other mechanism(s) leading to their loss of expression. The pleomorphic cells displayed increased proliferation (Ki-67) with atypical mitoses. The basal/myoepithelial cellular compartment was incomplete or lost, as p63, CK5, CK14, Calponin and α-SMA IHC demonstrated. These results implicate aberrant function in various pathways associated with amongst other ductal morphogenesis, epithelial cell lineage differentiation, and proliferation. Local and lymphovascular invasion were present (Figs. 2A, 3A and R), but the investigated lymph node samples were negative for ITC, micro- or macrometastases. The ECM showed modification (e.g., α-SMA IHC and Masson’s trichrome stain, Figs. 2C, K, 3C and K) and multifocal immune cell infiltration.

The total number of genomic variants was fivefold higher in the CC than in the SC. The number of non-silent variants (missense, stop-gain/loss, frameshift) was sixfold higher in the CC than in the SC. While 69% of exonic variants were non-silent in SC, 81% of exonic variants were non-silent in CC. The observed increase in the number of mutations, as well as the higher proportion of non-silent and pathogenic variants among the exonic variants, along with a greater number of driver genes in CC compared to SC, indicate a higher level of genomic instability in CC relative to SC [72, 73]. The subtypes did not share identical variants but had variants in 73 mutual genes suggesting intratumor heterogeneity. According to COSMIC, these 73 genes include cancer hallmark genes such as *NOTCH2* and *DICER1* with tumor suppressor gene (TSG) and oncogene (OG) function, *DNMT3A* and *FUS* with TSG function only, as well as the OGs *IKBKB*, *NSD3* and *NUP98*.

*NOTCH2* encodes a receptor of the Notch family, of which the members have temporally regulated function and expression in the development and maintenance of the normal mammary morphology and influence several hallmark genes in a cancer-specific manner, including in BC [74, 75]. Our subtypes carried different somatic variants of this gene: the SC, a probably damaging NSV and the CC, a stop-gain variant with loss-of-function, suggesting independent mutational incidence for each subtype. NOTCH2 expression increases in the normal mammary gland during lactation and after ovariectomy, implicating a hormonal dependency and role in luminal epithelial differentiation [76]. The CC carried another Notch receptor family variant: a probably damaging NSV in *NOTCH1*, a gene with TSG and OG function, and listed among the BC Tier 1 genes with annotated variants in COSMIC. *NOTCH1* mediates asymmetric cell division of mammary progenitor cells, resulting in self-renewal of stem cells [74]. In cell experiments, activation of NOTCH1 leads to luminal cell expansion, and its inhibition reduces stem cell population and mammosphere formation [74].

In the CC, a noteworthy stop-gain variant with loss-of-function located in *KRT15*. Cytoplasmic keratin intermediate filaments (KRT or CK) have essential intra- and intercellular functions with tissue and cell differentiation status-specific expression [77–79]. KRT15 positive epithelial cells in the basal layer are described as progenitor cells with contribution to tissue regeneration through the capacity of self-renewal and pluripotent epithelial lineage differentiation [80, 81]. In women KRT15 has been suggested as a marker to differentiate between DCIS and invasive BC, with a correlation of KRT15 expression with histological grade, tumor node stage and tumor node metastasis stage [82]. In invasive BC low KRT15 expression associates with poor prognosis [83]. To our knowledge, no previous reports exist on *KRT15* in the normal or tumorous canine mammary gland, and its expression and role need further exploration. Both investigated subtypes displayed an expansion of transformed cells of the luminal epithelial differentiation program with CK8, CK18 and CK19 expression as well as loss of the basally located stem cells expressing only CK5 without luminal or myoepithelial markers and of progenitor cells expressing CK5 or CK14 simultaneous with luminal or myoepithelial markers (Figs. 2E-G, I-J, 3E-G and I-J) [67]. As a result, we observed in both investigated subtypes the loss of functionally differentiated α-SMA^+^ myoepithelial cells (Figs. 2K and 3K) [67].

Basal/myoepithelial cells maintain tissue homeostasis by participating in BM production, mediation of luminal epithelial cell polarity and cell-to-BM attachment, as well as secretion of protease inhibitors, down-regulation of matrix metalloproteinases and paracrine decrease of angiogenesis [67, 84, 85]. In line with the observed changes in ductal morphology and size, we observed loss of the p63, CK5, CK14, α-SMA and Calponin expressing basal/myoepithelial cell populations (Figs. 2H-L and 3H-L). Interestingly, we did not identify somatic mutations in the corresponding genes (*TP63*, *KRT5*, *KRT14*, *ACTA2*, *CNN3*), suggesting possible modifications in upstream, reciprocal, or other regulatory components [67]. Loss of basal/myoepithelial cell markers and alterations in the otherwise strictly regulated BM and ECM composition and production facilitate aberrant luminal epithelial expansion, invasion, and metastasis [85–87]. In line with this, we identified in HE and Masson’s trichrome stains rudimentary BM and altered ECM, infiltrative growth and intravascular tumor cell emboli together with several SNVs in associated individual genes and the ECM-receptor interaction pathway, including various laminin (such as *LAMA1*, *LAMB1*, *LAMB2*, and *LAMB4*) and collagen subunits (*COL2A1*, *COL4A3*, *COL5A3*, *COL12A1*, *COL16A1* and C*OL22A1*), fibronectin (*FN1*), tenascin (*TNC*), thrombospondin (*THBS1*, *THBS3*), integrins (*ITGA2B*, *ITGA3*, *ITGA8*, *ITGA10* and *ITGB7*) and the ECM serine protease reelin (*RELN*) as well as the BM-specific heparan sulfate proteoglycan (*HSPG2*) (Figs. 2A, C, K, 3A, C, K and R; Tables S3B and S6B). These kind of genomic alterations were more abundant in CC compared to SC, despite close similarities in HE, Masson’s trichrome and α-SMA IHC stainings. The CC presented somatic variants in several subunit genes of laminin and collagen and the proteoglycans perlecan and versican. Of these, *LAMA1*, *LAMB1* and *LAMB4* showed loss-of-function frameshift deletions, *LAMB2*, *COL5A3*, *COL12A1* and *COL16A1* predicted benign NSVs, whereas *COL2A1* and C*OL22A1* probably damaging NSVs, and *VCAN* one benign and another probably damaging NSV.

*COL2A1* encodes the fiber-forming type II collagen, an ECM component providing the tissue tensile strength [88, 89]. Type II collagen fibrils join other collagen fibers and other ECM molecules through association with collagen type XII and link to the cellular cytoskeleton through integrins [88, 90]. Alteration of the normal ECM composition into carcinoma-associated ECM affects tissue homeostasis, tumorigenesis and metastatic progression [89]. COL2A1 is down-regulated in canine simple carcinoma-associated mammary stroma, and moderately expressed in canine simple carcinomas and up-regulated in canine complex carcinomas [91–93]. In BC primary tumors and cell lines up-regulation and down-regulation of COL2A1 are known, with down-regulation associated with migration and invasion [90, 91]. Furthermore, collagen type II acts as a ligand for the inhibitory leukocyte-associated immunoglobulin-like receptor-1 (LAIR-1), thus participating in immune response regulation [89].

We noticed only in the CC variants in the network-forming collagen IV family, which constitutes approximately 50% of the collagen in the BM of the mammary gland and provides it with structural support, functions as macromolecular diffusion barrier and exerts various collagen IV subtype-specific functions through integrin and non-integrin receptors, including the regulation of ERα expression and its function [87, 94–98]. Recently, BC subtype-specific expression patterns of collagen IV subtypes were suggested [98]. In our CC sample the collagen IV subtype *COL4A3* displayed two possibly damaging NSVs and one stop-gain mutation with loss-of-function. Interestingly, in the normal adult human mammary gland ducto-lobular BM expression of only COL4A1, COL4A2, COL4A5 and COL4A6 are demonstrated, but not that of COL4A3 [99, 100]. However, *COL4A3* has been identified as an angiogenesis-related gene and was recently determined as key prognostic gene in BC through its involvement in the regulation of epithelial cell differentiation and endothelial cell proliferation [101]. Additionally, expression of COL4A3 seems to correlate with a specific immune cell response in BC [101]. The loss of collagen IV subunit COL4A5 and COL4A6 expression has been demonstrated in invasive BC and their presence in the mammary BM is linked to functional α-SMA positive myoepithelial cells [100]. Our samples did not show alterations in *COL4A5* and *COL4A6*, but the observed loss of α-SMA positive myoepithelial cells in IHC in our samples could thus represent one further mechanism explaining the functional loss of the BM.

BM function is additionally affected by the observed NSVs of *LAMA3* and *HSPG2* which were predicted as probably damaging to the respective proteins in our CC. *LAMA3* encodes the alpha-3 subunit of laminin-5 (also laminin-332) in the mammary gland participating in cell adhesion (hemidesmosomes), migration and branching morphogenesis [102]. In BC, LAMA3 expression is partially or totally lost [103]. This can for example result following *LAMA3* promoter methylation as described in BC in association with increased tumor size and stage [104] or *LAMA3* mutations as previously described for inflammatory BC [105].

*HSPG2* encodes the protein perlecan which localizes in the normal mammary to the BM where it networks with collagen IV and laminin, to affect and participate in various functions such as cell–matrix adhesion, growth factor mediation, cell differentiation and angiogenesis [106]. In contrast to normal mammary, perlecan is fragmented or lost from the BM in BC and its synthesis translocated to stromal tumor cells and myofibroblasts facilitating stromal angiogenesis [106].

Additionally, CC had probably damaging NSVs in the matricellular protein genes *TNC*, *THBS1* and *THBS3*. TNC modulates epithelial cell interactions with the stromal ECM and is expressed in mammary epithelial cells and stroma [107]. Increased combined epithelial and stromal expression in BC associates with poorer prognosis and lymph node metastases [107, 108]. Similar expression is reported in CMTs [109–111]. TNC promotes tumor progression in several ways (e.g., metastasis and decreased immune response), and knock-down (KD) of TNC impaired BC metastasis and colonization to lungs and bone [112–114].

THBSs support calcium-dependent cell attachment, and *THBS1* is anti-angiogenetic and induces microvascular endothelial cell apoptosis, thus inhibiting tumor growth [115, 116]. However, in BC THBS1 is also reported to promote invasion and metastatic colonization through interactions with other proteins, and high expression is associated with poor prognosis [117–119]. *THBS3* is less investigated, and its significance remains to be explored.

The observed invasive nature could be further explained by alterations in focal and cell adhesion molecules. In our SC adherens junction genes such as *CREBBP*, *INSR*, *ACTN4* and *MAPK3* display NSVs with probable damaging pathogenicity, and *CSNK2B* has one frameshift deletion and one stop-loss NSV, both with loss-of-function. The CC shows variants in several genes related to ECM receptors, focal adhesion, axon guidance and cell adhesion pathways, of which some were already discussed. Interestingly, neither subtype had somatic mutations in *CDH1*, which encodes the transmembrane adherens junction protein E-Cadherin. However, the IHC expression pattern in the transformed intraluminal epithelial population was biphasic, with differences in peripheral compared to centrally located cells (Figs. 2D and 3D). Diagnostically, E-Cadherin positivity corresponds to the ductal origin of the carcinoma compared to the lobular origin, and variant expression patterns are associated with epithelial dedifferentiation, invasion and metastasis [66, 120, 121].

Disruption in cell–cell junction is a prerequisite for cell proliferation. Increased proliferative activity was HM examined in HE and using IHC against Ki-67 and determined as high score in the SC but intermediate in the CC (Figs. 2Q and 3Q). Atypical mitoses were present (Figs. 2B and 3B). These findings could correspond to genetic alterations in the CC pathways of base excision repair, apoptosis and actin cytoskeleton regulation. However, the HM observed differences in the proliferation activity in SC and CC need further exploration.

Likewise, MAPK pathway cascades are essential in the regulation of cellular processes such as differentiation, proliferation, survival, apoptosis as well as motility, and they comprise a multitude of sequential components [122–124]. In our material only the SC shows a NSV of *MAP3K1* with probably damaging pathogenicity. TSG and OG functions attribute to *MAP3K1*, which is also a Tier 1 BC gene [124]. Other genes in the MAPK signaling pathway show alterations in the investigated subtypes. *MAPK3* and *MAP4K4* had probably damaging NSVs in SC, and *MAP2K2* in CC. The CC displayed additionally benign NSVs in *MAP4K2* and *MAPKAPK5*, but loss-of-function in *MAP3K5* with a frameshift deletion and in *MAPK8IP3* a stop-gain variant.

Of other Tier 1 BC genes in COSMIC, the CC also carried variants in *ERBB2*, *NTRK3*, and *TBX3* with TSG and OG function, and in *NCOR1* with TSG function only. Two frameshift deletions with loss-of-function were identified in *NCOR1*, which encodes an estrogen receptor co-repressor and represents an independent prognostic factor in BC for disease-free and overall survival [125]. In their BC study, Zhang and colleagues observed lower NCOR1 mRNA expression with higher histological grade, negative ERα and PR statuses, but positive HER2 status and a tumor diameter larger than 2 cm [125]. This seems to align with our HM and IHC results.

*NTRK3* encodes TRKC, which activates the PI3K/Akt pathway, increases cell survival and prevents apoptosis [126]. *NTRK3* fusions are common in BC, but reports on other genomic alterations, such as copy number gain and mutation, are rare [126–129]. In CC we found one NSV that was predicted to be benign whereas no variations were seen in SC. To our knowledge, this is the first report of a NSV in canine *NTRK3*. A probably damaging NSV was determined in the CC *ERBB2*, encoding for HER2, a well-known BC biomarker with diagnostic, prognostic and therapeutic significance. Interestingly, our CC showed weak-to-moderate positive complete membrane reaction of HER2 in IHC in > 10% of the transformed luminal cells (Fig. 3O). However, it also is possible that our laser microdissection for WGS targeted a cluster of cells that display the observed NSV, indicating possible intrasubtype cellular heterogeneity, which could explain the observed HER2 IHC expression pattern. Contradicting data exists on HER2 expression in CMTs due to variations in definition of HER2-positive tumors and in applied biomarker panels for the identification of molecular subtypes [16, 130–135]. However, several SNPs are reported for the canine *ERBB2*, some associated with specific tumor subtypes [136, 137]. Furthermore, its loss is reported in malignant CMT [138]. Noteworthy, HER2 acts as a PIK3CA-Akt pathway activator [139].

*PIK3CA* is a significantly mutated driver gene and OG in BC and CMT, encoding a protein necessary for the enzyme PI3K [22–24, 135, 139–142]. A somatic variant in at least one PI3K pathway gene was reported in 50% of dogs with MTs [24]. Furthermore, Arendt and colleagues identified *PIK3CA* mutations in 25% of their investigated CMTs. These included a recurrent missense mutation homologous to hotspot mutation in various BC subtypes [139]. Although no specific alterations were found in *PIK3CA* in our subtypes, 51 genes involved in the PI3K-Akt pathway had NSVs in the CC, including the hallmark genes *BRCA1*, *ERBB2* and *COL2A1* with probably damaging prediction, as well as *MTOR* and *JAK3* with benign prediction, and *MYC* with a loss-of-function frameshift deletion.

Of these genes, the TSG and cell cycle regulator *BRCA1* is identified with germline and somatic mutations in BC [143, 144]. Likewise, various alterations of *BRCA1* are associated with CMT, especially with the loss of proper expression and function in malignant tumors [138, 139, 145–153]. Our CC showed an amino acid substitution and could possibly correspond with the reported cases with loss of BRCA1 expression in association with high Ki-67 expression and ER*α* negativity in IHC [145].

*MTOR* regulates multiple cellular events such as cell growth, homeostasis, survival and autophagy, cytoskeleton remodeling and cell motility through mTORC1 and mTORC2 complexes [154]. Usually, *MTOR* is dysregulated in cancer through upstream events, and only a few reports exist on human cancer-associated mutations in *MTOR* itself [155–157]. In BC, subtype differences are reported with activated p-mTOR localizing more often nuclear in triple-negative breast cancer (TNBC) than non-TNBC [158]. In previous canine studies, p-mTOR is not expressed in normal mammary tissues, but in 78% of mammary carcinomas, and the expression of the mTORC2 component Rictor is positively correlated with lymphatic invasion and poorer survival [159–161]. To our knowledge, no previous reports exist on somatic variants in the canine *MTOR*.

We determined one loss-of-function frameshift deletion in *MYC* in our CC. *MYC* is usually amplified in BC [162], with reports of amplification also in CMT [135, 138]. One *MYC* germline mutation with causative amino acid change of uncertain pathogenicity is reported in association with BC [163]. MYC has significant regulatory importance in cellular functions such as growth, differentiation, proliferation, apoptosis, and metabolism [162].

One NSV with benign prediction was determined in *JAK3* in our CC. Somatic mutations, amplifications, deep deletions and hypermethylation of *JAK3* exist in BC [164–166]. JAK3 is a member of the tyrosine kinase family with importance in cytokine signaling and plays a significant role in BC tumor cell invasion and transendothelial migration as well as an effector of a JAK3-distinct immune cell response [165, 167–169]. JAK3 regulates the PI3K-Akt pathway activation through association with IL-2 [166]. This is the first report of a NSV in *JAK3* associated with a CMT subtype.

Additionally, to the PI3K-Akt pathway, the CC displayed enrichment in the cGMP-PKG pathway with 25 affected genes, and predicted effects ranging from benign (e.g. *SRF* and *CREB3*), probably damaging (e.g. *MYLK*, *CREB5* and *MAP2K2*) to loss-of-function (e.g. *RAF1* and *PRKCE*). In BC cell culture studies, Fallahian and colleagues demonstrated that cGMP-PKG activation inhibits proliferation and induces apoptosis [170, 171]. PKG isoforms are downregulated in various BC subtypes [172]. Likewise, the apoptosis pathway of the CC was enriched with 21 affected genes, including *NFKB1* (predicted benign), *ACTB* and *FAS* (possibly damaging), *CTSB*, *MAP2K2* and *IKBKB* (probably damaging), as well as *RAF1* and *MAP3K5* (loss-of-function).

A further affected pathway in the SC was the NF-kappa-B pathway with several probably damaging variants (e.g., *TAB2*, *IL1R1* and *IKBKB*) and one loss-of-function variant (*ERC1*). The pathway has a significant role in the regulation of proliferation, apoptosis and inflammation, and activated NF-kappa-B is essential for a specific subset of ERα^−^ BC for proliferation and escapement of apoptosis [173, 174].

We also identified three cardiomyopathy-related pathways (dilated, hypertrophic and arrhythmogenic right ventricular) enriched in CC (Table S6B). A recent BC stratification study found dilated and hypertrophic cardiomyopathy pathways enriched together with other cancer related pathways [175]. The biological mechanisms and significance should be explored further.

Driver genes often promote tumor progression, and some are implicated prognostic in BC [176, 177]. While drivers are increasingly identified in BC, their exploration in CMT remains low, with previous reports of only *PIK3CA* as such [22, 142]. We predicted *NEO1*, *ATP6V1C2*, *GLYATL3*, *CARMIL3*, *GATAD2B*, *OBSCN*, *SIX2*, *CPEB3* and *ZNF521* as drivers. The TSG *NEO1* was identified in both subtypes, and displayed a loss-of-function stop-gain NSV in the SC and a probably damaging NSV in the CC. The cell adhesion molecule Neogenin-1 is significant in mammary morphogenesis together with the laminin-related Netrin-1 [178, 179]. Decreased or lost neogenin expression is reported in BC [179]. Loss of the Netrin-1/Neogenin complex is associated with aberrant ductal morphogenesis and breaks in the BM [178]. Furthermore, NEO1 plays a role in tumor progression, angiogenesis, apoptosis and migration in colorectal cancer and neuroblastoma cells [180, 181].

The V-ATPase-enzyme subunit gene *ATP6V1C2* was predicted as driver only in the SC and displayed a probably damaging NSV. Differences in expression statuses and prognostic significance are reported in various cancer cell lines, with a less significant role in BC, present in 7% of cases by overexpression or genomic amplification [182, 183]. In a colon adenocarcinoma study, ATP6V1C2 knock-down (KD) affected WNT pathway-related genes and epithelial-to-mesenchymal transition (EMT)-related genes, with a decrease in fibronectin-1, vimentin and increase in E-Cadherin, thus attenuating metastasis [182].

Of the predicted drivers in CC, a probably damaging NSV was demonstrated in *GATAD2B*, a transcription factor and essential component of the Nucleosome Remodelling and Deacetylation (NuRD) complex that regulates gene expression through chromatin remodeling, and thus plays a role in transcription regulation, chromatin assembly, genomic stability and cell cycle progression [184–187]. Depending on the cellular context, NuRD can promote or suppress tumorigenesis, and through the complex members MTA1 and MTA2 affect tumor progression and metastasis [186, 188]. One paper identified GATAD2B to enhance tumor growth through direct interaction with MYC [188]. However, the specific role of *GATAD2B* in cancers is poorly investigated.

CC harbored a probably damaging NSV in the predicted driver *CPEB3*. CPEBs regulate cytoplasmic translation through mRNA binding [189]. Data on *CPEB3* in the mammary gland and BC is limited [190]. *CPEB3* is suggested as TSG with implications of regulatory involvement in various pathways associated with tumor progression [189, 191]. Down-regulation in digestive tract cancers is demonstrated, and for example CPEB3 KD increased proliferation, migration and invasion in colorectal cancer cells via the JAK/STAT pathway activation [189].

One NSV, predicted to be benign, was detected in CC *CARMIL3*. *CARMIL3* regulates cytoskeletal actin organization, actin-based mobility, and via ZEB2 the expression of E-Cadherin, with *CARMIL3* loss leading to attenuated focal adhesions, cellular polarization as well as impaired cellular migration, invasion and metastatic colonization [192].

Of the predicted other drivers, *SIX2* regulates BC metastasis by downregulating E-Cadherin, and KD of SIX2 decreases distant metastasis without affecting primary tumor growth or the associated (lymph)angiogenesis [193]. Our CC displayed a possibly damaging NSV. *OBSCN* is frequently mutated in BC and is implicated as TSG with cell morphology, adhesion and migration functions, preventing EMT and metastasis [194–196]. Our CC presented with two probably damaging NSVs. Expression of the stem cell-associated transcription co-factor ZNF521 associates with a protective role in invasive BC [197]. Its aberrant expression is reported in several cancers [198]. Our CC displayed a benign NSV. The role of the enzyme GLYATL3 in cancer is less explored [199, 200]. The relevance of the probably damaging NSV in the CC remains to be investigated. Following Li and colleagues, our results suggest that some driver genes might be subtype-specific and accumulate with increasing malignancy [142].

Unlike previous CMT studies, we did not find any variation in *TP53*, but our CC had NSV and frameshift deletion variants in *TP53RK* and *TP53BP1*, respectively [135, 138, 201, 202]. Both play a role in p53 activation [203–205]. Furthermore, loss of *TP53BP1* function increases angiogenesis and is linked to poor prognosis in cancer [205]. Nor did we identify mutations in *PTEN*, which are previously reported for benign and malignant CMTs [137–139].

Our methodological approach allowed precision comparison between transformed ductal luminal epithelial cells from two intratumoral phenotypic distinct subtype components within a single canine individual, and to investigate their HM, immunophenotypical and genomic relationships. We demonstrated differences between the subtypes and correlation of HM and IHC with the identified genomic alterations, but also observed changes in IHC without modifications in the respective genes. The number of shared mutations in the SC and CC samples was relatively low, with CC displaying a higher overall mutation frequency. Also, the pathways involved differed significantly between the subtypes, as did the specific mutations in the genes that were mutated in both samples, inferring differences in clinical behavior. For example, genes affected in CC were often correlated to BM function and ECM composition, indicating possibly a higher ability to escape and metastasize. Interestingly, neither of the subtypes displayed mutations in the basal/myoepithelial biomarkers *TP63*, *KRT5*, *KRT14*, *ACTA2* and *CNN3* despite their loss of expression in IHC (p63, CK5, CK14, α-SMA, Calponin). Nor did we detect any mutations in the genes encoding the hormone receptors ERα (*ESR1*) and PR (*PGR*) but identified several ways in which the expression of ERα could be affected. These results remind us of the complexity of interdependencies in the expression of cellular components and the diversity of possible genetic, epigenetic and transcriptomic alterations in cancer.

With our sample material it is difficult to determine if the observed intratumor heterogeneity could indicate an independent origin for the subtypes. Intratumor heterogeneity is taken into consideration in the human classification system with the category of NST tumors with mixed histological pattern [48]. In the dog, intratumor heterogeneity has been described but to our knowledge its frequency has not been further investigated. We acknowledge the limitations of only investigating subtypes from a single dog and the low sequencing depths accomplished with respect to our findings. Investigation of additional subtype-specific cell clusters and their microenvironment with known spatial location in this kind of heterogenic tumor would improve our insight of intratumor heterogeneity, clonal expansion and tumor evolution [72, 73, 206–208]. Moreover, functional cell culture studies would assess the relevance and significance of our observations. Alteration in IHC biomarker expression can be a direct effect of gene modification or result from changes in complex cell regulatory events. Furthermore, many of our findings suggest significant alterations to the BM and ECM. Tumorigenesis, progression, invasion and metastasis are complex and multifactorial. Complex reciprocal interactions between transformed epithelial cells and tumor microenvironment provoke further exploration and characterization into subtype-specific pathogenesis and progression as the basis for individualized precision medicine. We did not have tissue samples from equivalent BC subtypes to analyze, but we used available literature and databases such as COSMIC and KEGG to detect subset overlap with BC. Finally, our data demonstrate the importance of thoroughly characterizing research specimens suggested for translational cancer studies. However, further exploration with a larger CMT sample size and comprehensive cell culture studies are needed to verify our findings.

## Conclusions

We demonstrated differential coding variant landscapes and pathway affections between the canine invasive SC and CC ductal luminal epithelial cells of a mixed phenotype CMT with immunophenotypic similarities. We identified new driver genes with probable subtype specificity. Furthermore, we uncovered subtype molecular mechanism profiles also affecting BM and ECM homeostasis. Significantly, some involved genes and pathways overlapped with BC data indicating a potential use of CMT as model for BC for certain subtypes. Additionally, we observed alterations in biomarker IHC expression without respective genetic mutations, indicating changes to their complex signaling pathways, disturbed regulative feedback loops or other silencing mechanisms. This demonstrates the continuing significance of IHC as a clinical diagnostic tool. In all, our data support subtype-distinct molecular biological disease entities and intensify the need to develop individualized subtype-specific diagnostic and therapeutic intervention methods. Our data are essential for translational research and are worth further exploration and validation using larger sample sizes and cell cultural experiments of humans and canines.

## Supplementary Information

Additional file 1. Table S1. List of applied IHC antibodies.

Additional file 2. Figure S1 Preseq2 complexity estimation and yield prediction for PicoPLEX libraries. Black line in A–C represents standard library, and colored lines low-coverage libraries sequenced at 1x. A Normal lobular. B Solid carcinoma. C Comedocarcinoma. Both normal sample libraries (IDs DOG_MAMMA_28 and DOG_MAMMA_32), one solid carcinoma sample library (ID DOG_MAMMA_1), and one comedocarcinoma sample library (ID DOG_MAMMA_10) were selected for further sequencing.

Additional file 3. Figure S2 IGV (Integrative Genome Viewer) coverage plot for gene *ERBB2* (NM_001003217) and region (chr9:22,713,320–22,736,532). An increase in coverage of reads from the BAM file is observed in solid carcinoma (ID DOG_MAMMA_1) and comedocarcinoma (ID DOG_MAMMA_10) samples compared to the two normal samples (IDs DOG_MAMMA_28 and DOG_MAMMA_32) in regions chr9:22,723,000–22,725,00.

Additional file 4. Figure S3 Histomorphology and biomarker immunohistochemistry of normal canine mammary gland. Structures have been annotated as follows: S=stroma, L=lobule, asterix=duct. A Normal ductolobular morphology. HE. Scale bar 100 μm. B Ductolobular units red, collagenous basement membrane and fibrovascular stroma blue. Masson’s trichrome. Scale bar 100 μm. C High epithelial E-Cadherin expression. Scale bar 50 μm. Counterstain Harris hematoxylin. D High luminal epithelial CK8 expression. Scale bar 50 μm. Counterstain Harris hematoxylin. E Weak-to-moderate luminal epithelial CK19 expression. Scale bar 50 μm. Counterstain Harris hematoxylin. F High luminal epithelial CK18 expression. Scale bar 50 μm. Counterstain Harris hematoxylin. G High basal/ myoepithelial nuclear p63 expression. Scale bar 50 μm. Counterstain Harris hematoxylin. H High basal / myoepithelial CK5 expression. Scale bar 100 μm. Counterstain Harris hematoxylin. I High basal / myoepithelial CK14 expression. Scale bar 50 μm. Counterstain Harris hematoxylin. J High basal / myoepithelial and vascular α-SMA expression. Occasional stromal myofibroblasts are positive. Scale bar 100 μm. Counterstain Harris hematoxylin. K High basal / myoepithelial Calponin expression. Occasional stromal myofibroblasts are positive. Scale bar 50 μm. Counterstain Harris hematoxylin. L Weak-to-moderate nuclear epithelial ERα expression. Scale bar 50 μm. Counterstain Harris hematoxylin. M Moderate-to-high nuclear epithelial PR expression. Scale bar 50 μm. Counterstain Harris hematoxylin. N High membranous (and cytoplasmic) HER2 expression adjacent to investigated tumor mass. Scale bar 50 μm. Counterstain Harris hematoxylin. O Weak-to-moderate membranous EGFR expression. Scale bar 50 μm. Counterstain Harris hematoxylin. P Occasional nuclear epithelial Ki-67 expression. Scale bar 50 μm. Counterstain Harris hematoxylin.

Additional file 5. Table S2. Amplifications and deletions identified by VarScan 2 in canine invasive ductal solid carcinoma and comedocarcinoma compared with normal samples. Table S3A. Somatic variants and predicted pathogenicity in canine invasive ductal. solid carcinoma. Table S3B. Somatic variants and predicted pathogenicity in canine invasive ductal comedocarcinoma. Table S4A. Structural variants in canine invasive ductal solid carcinoma. Table S4B. Structural variants in canine invasive ductal. comedocarcinoma. Table S5. Mutual genes with variants in canine invasive ductal solid carcinoma and comedocarcinoma. Table S6A. KEGG pathways in canine invasive ductal solid carcinoma. Table S6B. KEGG pathways in canine invasive ductal. comedocarcinoma. Table S7. Driver genes identified by OncodriveCLUSTL from canine invasive ductal solid carcinoma and comedocarcinoma. Table S8. List of genes with non-silent variants in canine invasive ductal solid carcinoma and comedocarcinoma compared with COSMIC-CGC and KEGG databases.

## Data Availability

The whole genome sequencing datasets generated and analysed from two canine mammary normal lobular libraries, one simple solid carcinoma and one comedocarcinoma subtypes have been submitted to NCBI SRA BioProject – PRJNA913118 and SRA accession IDs SRR22765699, SRR22765700, SRR22765701 and SRR22765702. The samples to create PON and in-house canine database are added to the NCBI SRA BioProject PRJNA913118.

## Abbreviations

α-SMA: Alpha-Smooth muscle actin
Asterix: Necrosis (Fig. 3) and duct (Figure S3)
BAM: Binary Alignment Map
BC: Human breast cancer
CC: Comedocarcinoma
CK: Cytokeratine
CMT: Canine mammary tumor
COSMIC-CGC: Catalogue of Somatic Mutations in Cancer, Cancer Gene Census
DAB: 3.3´-Diaminobenzidine-tetrahydrochloride
DAVID: Database for Annotation, Visualization and Integrated Discovery
DNA: Deoxyribonucleic acid
EGFR: Epidermal growth factor receptor
EMT: Epithelial-to-mesenchymal transition
ER: Estrogen receptor
FFPE: Formalin-fixed paraffin-embedded
HER2: Human epidermal growth factor receptor-2
HM: Histomorphology
IGV: Integrative Genomics Viewer
IHC: Immunohistochemistry
ITC: Isolated tumor cells
KD: Knock-down
KEGG: Kyoto Encyclopedia of Genes and Genomes
KRT: Keratine
NGS: Next generation sequencing
L: Lobule (Figure S3)
LE: Luminal epithelium (Figs. 2 and 3)
NST: No special type
LCM: Laser-capture microdissection
NSV: Non-synonymous variant
OG: Oncogene
PBS: Phosphate-buffered saline
PON: Panel of Normal
PR: Progesterone receptor
pTNM: Pathologic stage comprising tumor extent, regional lymph node status and distant metastasis
RT: Room temperature
S: Stroma (Figs. 2, 3 and S3)
SNP: Single Nucleotide Polymorphism
SNV: Single nucleotide variant
SS: Solid carcinoma
SV: Structural variant
TBS: Tris-buffered saline
TSG: Tumor suppressor gene
TNBC: Triple-negative breast cancer
WGS: Whole genome sequencing

## References

[1] Sung H, Ferlay J, Siegel RL, Laversanne M, Soerjomataram I, Jemal A, et al. Global Cancer Statistics 2020: GLOBOCAN estimates of incidence and mortality worldwide for 36 cancers in 185 countries. CA Cancer J Clin. 2021;71:209–49. 33538338 10.3322/caac.21660

[2] Sabatier R, Gonçalves A, Bertucci F. Personalized medicine: Present and future of breast management. Crit Rev Oncol Hematol. 2014;91:223–33. 24725667 10.1016/j.critrevonc.2014.03.002

[3] Tomczak K, Czerwínska P, Wiznerowicz M. The Cancer Genome Atlas (TCGA): An immeasurable source of knowledge. Contemp Oncol (Pozn). 2015;19:A68–77. 25691825 10.5114/wo.2014.47136 PMC4322527

[4] Sondka Z, Bamford S, Cole CG, Ward SA, Dunham I, Forbes SA. The COSMIC Cancer Gene Census: describing genetic dysfunction across all human cancers. Nat Rev Cancer. 2018;18:696–705. 30293088 10.1038/s41568-018-0060-1 PMC6450507

[5] Tsuchida J, Rothman J, McDonald KA, Nagahashi M, Takabe K, Wakai T. Clinical target sequencing for precision medicine of breast cancer. Int J Clin Oncol. 2019;24:131–40. 30604156 10.1007/s10147-018-1373-5

[6] Yang Y, Dong X, Xie B, Ding N, Chen J, Li Y, Zhang Q, Qu H, Fang X. Databases and web tools for cancer genomics study. Genomics Proteomics Bioinformatics. 2015;13:46–50. 25707591 10.1016/j.gpb.2015.01.005 PMC4411507

[7] Arnedo-Pac C, Mularoni L, Muiños F, Gonzalez-Perez A, Lopez-Bigas N. OncodriveCLUSTL: a sequence-based clustering method to identify cancer drivers. Bioinformatics. 2019;35:4788–90. 31228182 10.1093/bioinformatics/btz501 PMC6853674

[8] Lee YT, Tan YJ, Oon CE. Molecular targeted therapy: Treating cancer with specifity. Eur J Pharmacol. 2018;834:188–96. 30031797 10.1016/j.ejphar.2018.07.034

[9] Peña L, Gama A, Goldschmidt MH, Abadie J, Benazzi C, Castagnaro M, et al. Canine mammary tumors: a review and consensus of standard guidelines on epithelial and myoepithelial phenotype markers, HER2, and hormone receptor assessment using immunohistochemistry. Vet Pathol. 2014;51:127–45. 24227007 10.1177/0300985813509388

[10] Vascellari M, Capello K, Carminato A, Zanardello C, Baioni E, Mutinelli F. Incidence of mammary tumors in the canine population living in the Veneto region (Northeastern Italy): Risk factors and similarities to human breast cancer. Prev Vet Med. 2016;126:183–9. 26948297 10.1016/j.prevetmed.2016.02.008

[11] Schneider R. Comparison of age, sex, and incidence rates in human and canine breast cancer. Cancer. 1970;26:419–26. 5465470 10.1002/1097-0142(197008)26:2<419::aid-cncr2820260225>3.0.co;2-u

[12] Millanta F, Calandrella M, Bari G, Niccolini M, Vannozzi I, Poli A. Comparison of steroid receptor expression in normal, dysplastic, and neoplastic canine and feline mammary tissues. Res Vet Sci. 2005;79:225–32. 16054892 10.1016/j.rvsc.2005.02.002

[13] Gama A, Alves A, Schmitt F. Identification of molecular phenotypes in canine mammary carcinomas with clinical implications: application of the human classification. Virchows Arch. 2008;453:123–32. 18677512 10.1007/s00428-008-0644-3

[14] Sassi F, Benazzi C, Castellani G, Sarli G. Molecular-based tumour subtypes of canine mammary carcinomas assessed by immunohistochemistry. BMC Vet Res. 2010;6:5. 20109214 10.1186/1746-6148-6-5 PMC2837647

[15] Sorenmo KU, Rasotto R, Zappulli V, Goldschmidt MH. Development, anatomy, histology, lymphatic drainage, clinical features, and cell differentiation markers of canine mammary gland neoplasms. Vet Pathol. 2011;48:85–97. 21147765 10.1177/0300985810389480

[16] Im KS, Kim NH, Lim HY, Kim HW, Shin JI, Sur JH. Analysis of a new histological and molecular-based classification of canine mammary neoplasia. Vet Pathol. 2014;51:549–59. 24003019 10.1177/0300985813498780

[17] Abadie J, Nguyen F, Loussouarn D, Peña L, Gama A, Rieder N, et al. Canine invasive mammary carcinomas as models of human breast cancer. Part 2: Immunophenotypes and prognostic significance. Breast Cancer Res Treat. 2018;167:459–68. 29063312 10.1007/s10549-017-4542-8 PMC5790838

[18] Valdivia G, Alonso-Diez À, Pérez-Alenza D, Peña L. From conventional to precision therapy in canine mammary cancer: A comprehensive review. Fron Vet Sci. 2021;8:623800. 10.3389/fvets.2021.623800 PMC7925635 33681329

[19] Gray M, Meehan J, Martinez-Pérez C, Kay C, Turnbull AK, Morrison LR, et al. Naturally-occurring canine mammary tumors as a translational model for human breast cancer. Front Oncol. 2020;10:617. 32411603 10.3389/fonc.2020.00617 PMC7198768

[20] Beck J, Hennecke S, Bornemann-Kolatzki K, Urnovitz HB, Neumann S, Ströbel P, et al. Genome aberrations in canine mammary carcinomas and their detection in cell-free plasma DNA. PLoS ONE. 2013. 10.1371/journal.pone.0075485. 24098698 10.1371/journal.pone.0075485 PMC3787092

[21] Liu D, Xiong H, Ellis AE, Northrup NC, Rodrìguez CO Jr, O’Regan RM, et al. Molecular homology and difference between spontaneous canine mammary cancer and human breast cancer. Cancer Res. 2014;74:5045–56. 25082814 10.1158/0008-5472.CAN-14-0392 PMC4167563

[22] Lee KH, Hwang HJ, Noh HJ, Shin TJ, Cho JY. Somatic mutation of *PIK3CA* (H1047R) is a common driver mutation hotspot in canine mammary tumors as well as human breast cancers. Cancers (Basel). 2019;11:2006. 31842489 10.3390/cancers11122006 PMC6966585

[23] Kim TM, Yang IS, Seung BJ, Lee S, Kim D, Ha YJ, et al. Cross-species oncogenic signatures of breast cancer in canine mammary tumors. Nat Commun. 2020;11:3616. 32680987 10.1038/s41467-020-17458-0 PMC7367841

[24] Alsaihati BA, Ho K-L, Watson J, Feng Y, Wang T, Dobbin KK, et al. Canine tumor mutational burden is correlated with TP53 mutation across tumor types and breeds. Nat Commun. 2021;12:4670. 34344882 10.1038/s41467-021-24836-9 PMC8333103

[25] Goldschmidt M, Peña L, Rasotto R, Zappulli V. Classification and grading of canine mammary tumors. Vet Pathol. 2011;48:117–31. 21266722 10.1177/0300985810393258

[26] Nakagaki YR, Nunes MM, Garcia APV, Nunes FC, Schmitt F, Cassali GD. Solid carcinoma of the canine mammary gland: A histological type or tumour cell arrangement? J Comp Path. 2022;190:1–12. 35152966 10.1016/j.jcpa.2021.10.011

[27] Lakhani, SR. IARC & WHO Classification of Tumours Editorial Board. WHO classification of breast tumours. World Health Organization classification of tumours. Lyon: IARC. 2019. p. 368.

[28] Cramer H. Cytopathology of metastatic breast cancer. Clin Breast Cancer. 2000;1:243–4. 11899649 10.3816/CBC.2000.n.021

[29] Yagata H, Harigaya K, Suzuki M, Nagashima T, Hashimoto H, Ishii G, et al. Comedonecrosis is an unfavorable marker in node-negative invasive breast carcinoma. Pathol Int. 2003;53:501–6. 12895228 10.1046/j.1440-1827.2003.01514.x

[30] Kordek R. Ductal carcinoma in situ-like structures in metastatic breast carcinoma. Pathol Res Pract. 2005;200:831–4. 15792128 10.1016/j.prp.2004.08.006

[31] Pervez S, Khan H. Infiltratring ductal carcinoma breast with central necrosis closely mimicking ductal carcinoma in situ (comedo type): A case series. J Med Case Reports. 2007;1:83. 10.1186/1752-1947-1-83 PMC2014768 17825107

[32] Coyne J. Metastatic mammary carcinoma with DCIS-like morphology: A report of two cases. Int J Surg Path. 2012;20:485–7. 22297833 10.1177/1066896911435724

[33] Mohan N, Black JO, Schwartz MR, Zhai QJ. Invasive ductal carcinoma with in situ pattern: How to avoid this diagnostic pitfall? Am J Transl Res. 2016;8:3337–41. 27648124 PMC5009386

[34] Rosen PP. Rosen´s Breast Pathology. New York: Lippincott-Raven, 2009. p. 285.

[35] Rasotto R, Zappulli V, Castagnaro M, Goldschmidt MH. A retrospective study of those histopathologic parameters predictive of invasion of the lymphatic system by canine mammary carcinomas. Vet Pathol. 2012;49:330–40. 21670194 10.1177/0300985811409253

[36] Rasotto R, Berlato D, Goldschmidt MH, Zappulli V. Prognostic significance of canine mammary tumor histologic subtypes: An observational cohort study of 229 cases. Vet Pathol. 2017;54:571–8. 28355108 10.1177/0300985817698208

[37] Peña L, De Andrés PJ, Clemente M, Cuesta P, Pérez-Alenza MD. Prognostic value of histological grading in noninflammatory canine mammary carcinomas in a prospective study with two-year follow-up: Relationship with clinical and histological characteristics. Vet Pathol. 2013;50:94–105. 22688585 10.1177/0300985812447830

[38] Mainenti M, Rasotto R, Carnier P, Zappulli V. Oestrogen and progesterone receptor expression in subtypes of canine mammary tumours in intact and ovariectomised dogs. Vet J. 2014;202:62–8. 24980810 10.1016/j.tvjl.2014.06.003

[39] Yoshimura H, Nakahira R, Kishimoto TE, Michishita M, Ohkusu-Tsukada K, Takahashi K. Differences in indicators of malignancy between luminal epithelial cell type and myoepithelial cell type of simple solid carcinoma in the canine mammary gland. Vet Pathol. 2014;51:1090–5. 24448671 10.1177/0300985813516637

[40] Canadas A, França M, Pereira C, Vilaça R, Vilhena H, et al. Canine mammary tumors: Comparison of classification and grading methods in a survival study. Vet Pathol. 2019;56:208–19. 30381007 10.1177/0300985818806968

[41] Seung B, Cho S, Kim S, Bae M, Lim H, Kwak S, et al. Impact of histological subtype on survival in canine mammary carcinomas: A retrospective analysis of 155 cases. J Comp Path. 2021;186:23–30. 34340801 10.1016/j.jcpa.2021.05.002

[42] Kamstock DA, Ehrhart EJ, Getzy DM, Bacon NJ, Rassnick KM, Moroff SD, et al. Recommended guidelines for submission, trimming, margin evaluation, and reporting of tumor biopsy specimens in veterinary surgical pathology. Vet Pathol. 2011;48:19–31. 21123864 10.1177/0300985810389316

[43] Lester SC, Bose S, Chen Y, Connolly J, de Baca ME, Fitzgibbons PL, et al. Protocol for the examination of specimens from patients with invasive carcinoma of the breast. Arch Pathol Lab Med. 2009;133:1515–38. 19792042 10.5858/133.10.1515

[44] Edge SB, Byrd DR, Compton CC, Fritz AG, Greene FL, Trotti A, editors. AJCC Cancer Staging Manual. 7th ed. New York: Springer; 2010.

[45] Moriya T, Kozuka Y, Kanomata N, Tse GM, Tan PH. The role of immunohistochemistry in the differential diagnosis of breast lesions. Pathology. 2009;41:68–76. 19089742 10.1080/00313020802563544

[46] Nakahira R, Michishita M, Yoshimura H, Hatakeyama H, Takahashi K. Neuroendocrine carcinoma of the mammary gland in a dog. J Comp Path. 2015;152:188–91. 25670668 10.1016/j.jcpa.2014.12.009

[47] Nakagaki KYR, Nunes MM, Vargas Garcia AP, De Brot M, Cassali GD. Neuroendocrine carcinomas of the canine mammary gland: Histopathological and immunohistochemical characteristics. Front Vet Sci. 2021;7: 621714. 33469557 10.3389/fvets.2020.621714 PMC7813755

[48] Rakha EA, Masuda S, Allison KH, Penault-Llorca F, Bu H, Schnitt SJ, et al. Invasive breast carcinoma of no special type. In: WHO Classification of Tumours Editorial Board. Breast Tumours. WHO classification of tumours series, 5^th^ ed.; vol. 2. Lyon: International Agency for Research on Cancer; 2019. p. 102–9.

[49] Dowsett M, Nielsen TO, A’Hern R, Bartlett J, Coombes RC, Cuzick J, International Ki-67 in Breast Cancer Working Group., et al. Assessment of Ki67 in breast cancer: recommendations from the International Ki67 in Breast Cancer working group. J Natl Cancer Inst. 2011;103:1656–64. 21960707 10.1093/jnci/djr393 PMC3216967

[50] Hammond ME, Hayes DF, Dowsett M, Allred DC, Hagerty KL, Badve S, et al. American Society of Clinical Oncology/College Of American Pathologists guideline recommendations for immunohistochemical testing of estrogen and progesterone receptors in breast cancer. J Clin Oncol. 2010;28:2784–95. 20404251 10.1200/JCO.2009.25.6529 PMC2881855

[51] Duffy MJ, Harbeck N, Nap M, Molina R, Nicolini A, Senkus E, et al. Clinical use of biomarkers in breast cancer: Updated guidelines from the European Group on Tumor Markers (EGTM). Eur J Cancer. 2017;75:284–98. 28259011 10.1016/j.ejca.2017.01.017

[52] Wolff AC, Hammond MEH, Allison KH, Harvey BE, Mangu PB, Bartlett JMS, et al. Human epidermal growth factor receptor 2 testing in breast cancer: American Society of Clinical Oncology/College of American pathologists clinical practice guideline focused update. Arch Pathol Lab Med. 2018;142:1364–82. 29846104 10.5858/arpa.2018-0902-SA

[53] Nielsen TO, Leung SCY, Rimm DL, Dodson A, Acs B, Badve S, et al. Assessment of Ki67 in Breast Cancer: Updated Recommendations From the International Ki67 in Breast Cancer Working Group. J Natl Cancer Inst. 2021;113:808–19. 33369635 10.1093/jnci/djaa201 PMC8487652

[54] Daley T, Smith AD. Predicting the molecular complexity of sequencing libraries. Nat Methods. 2013;10:325–7. 23435259 10.1038/nmeth.2375 PMC3612374

[55] Wang C, Wallerman O, Arendt ML, Sundström E, Karlsson Å, Nordin J, et al. A novel canine reference genome resolves genomic architecture and uncovers transcript complexity. Commun Biol. 2021;4:185. 33568770 10.1038/s42003-021-01698-x PMC7875987

[56] Cibulskis K, Lawrence MS, Carter SL, Sivachenko A, Jaffe D, Sougnez C, et al. Sensitive detection of somatic point mutations in impure and heterogeneous cancer samples. Nat Biotechnol. 2013;31:213–9. 10.1038/nbt.2514 PMC3833702 23396013

[57] Rausch T, Zichner T, Schlattl A, Stütz AM, Benes V, Korbel JO. DELLY: structural variant discovery by integrated paired-end and split-read analysis. Bioinformatics. 2012;28:i333–9. 22962449 10.1093/bioinformatics/bts378 PMC3436805

[58] Thorvaldsdóttir H, Robinson JT, Mesirov JP. Integrative Genomics Viewer (IGV): high-performance genomics data visualization and exploration. Brief Bioinform. 2013;14:178–92. 22517427 10.1093/bib/bbs017 PMC3603213

[59] Nattestad M, Aboukhalil R, Chin CS, Schatz MC. Ribbon: intuitive visualization for complex genomic variation. Bioinformatics. 2021;37:413–5. 32766814 10.1093/bioinformatics/btaa680 PMC8058763

[60] Wang K, Li M, Hakonarson H. ANNOVAR: functional annotation of genetic variants from high-throughput sequencing data. Nucleic Acids Res. 2010. 10.1093/nar/gkq603. 20601685 10.1093/nar/gkq603 PMC2938201

[61] Adzhubei IA, Schmidt S, Peshkin L, Ramensky VE, Gerasimova A, Bork P, et al. A method and server for predicting damaging missense mutations. Nat Methods. 2010;7:248–9. 20354512 10.1038/nmeth0410-248 PMC2855889

[62] Adzhubei I, Jordan DM, Sunyaev SR. Predicting functional effect of human missense mutations using PolyPhen-2. Curr Protoc Hum Genet. 2013. 10.1002/0471142905.hg0720s76. 23315928 10.1002/0471142905.hg0720s76 PMC4480630

[63] Koboldt DC, Zhang Q, Larson DE, Shen D, McLellan MD, Lin L, et al. VarScan 2: somatic mutation and copy number alteration discovery in cancer by exome sequencing. Genome Res. 2012;22:568–76. 22300766 10.1101/gr.129684.111 PMC3290792

[64] Kinsella RJ, Kahari A, Haider S, Zamora J, Proctor G, Spudich G, et al. Ensembl BioMarts: a hub for data retrieval across taxonomic space. Database. 2011;2011:bar030. 21785142 10.1093/database/bar030 PMC3170168

[65] Tate JG, Bamford S, Jubb HC, Sondka Z, Beare DM, Bindal N, et al. COSMIC: the Catalogue of somatic mutations in cancer. Nucleic Acids Res. 2019;47(D1):D941–7. 30371878 10.1093/nar/gky1015 PMC6323903

[66] Moll R, Mitze M, Frixen UH, Birchmeier W. Differential loss of E-cadherin expression in infiltrating ductal and lobular breast carcinomas. Am J Pathol. 1993;143:1731–42. 8256859 PMC1887260

[67] Deckwirth V, Rajakylä EK, Cattavarayane S, Acheva A, Schaible N, Krishnan R, et al. Cytokeratin 5 determines maturation of the mammary myoepithelium. iScience. 2021;24:102413. 34007958 10.1016/j.isci.2021.102413 PMC8111680

[68] Chocteau F, Abadie J, Loussouarn D, Nguyen F. Proposal for a histological staging system of mammary carcinomas in dogs and cats part 1: canine mammary carcinomas. Front Vet Sci. 2019;6:388. 31788485 10.3389/fvets.2019.00388 PMC6854021

[69] Sorlie T, Tibshirani R, Parker J, Hastie T, Marron JS, Nobel A, et al. Repeated observation of breast tumor subtypes in independent gene expression data sets. Proc Natl Acad Sci U S A. 2003;100:8418–23. 12829800 10.1073/pnas.0932692100 PMC166244

[70] KEGG PATHWAY Database. http://tumor.informatics.jax.org/cancer_links.html. Accessed 30 Jan 2023.

[71] Catalogue Of Somatic Mutations In Cancer (COSMIC). The Cancer Gene Census. https://cancer.sanger.ac.uk/census. Accessed 19 Jan 2023.

[72] Burrell RA, McGranahan N, Bartek J, Swanton C. The causes and consequences of genetic heterogeneity in cancer evolution. Nature. 2013;501:338–45. 24048066 10.1038/nature12625

[73] McGranahan N, Swanton C. Clonal Heterogeneity and Tumor Evolution: Past, Present, and the Future. Cell. 2017;168:613–28. 28187284 10.1016/j.cell.2017.01.018

[74] Park JP, Raafat A, Feltracco JA, Blanding WM, Booth BW. Differential gene expression in nuclear label-retaining cells in the developing mouse mammary gland. Stem Cells Dev. 2013;22:1297–306. 23199335 10.1089/scd.2012.0496

[75] Aster JC, Pear WS, Blacklow SC. The Varied Roles of Notch in Cancer. Annu Rev Pathol. 2017;12:245–75. 27959635 10.1146/annurev-pathol-052016-100127 PMC5933931

[76] Raafat A, Goldhar AS, Klauzinska M, Xu K, Amirjazil I, McCurdy D, et al. Expression of Notch receptors, ligands, and target genes during development of the mouse mammary gland. J Cell Physiol. 2011;226:1940–52. 21506125 10.1002/jcp.22526 PMC3073161

[77] Moll R, Franke WW, Schiller DL, Geiger B, Krepler R. The catalog of human cytokeratins: patterns of expression in normal epithelia, tumors and cultured cells. Cell. 1982;31:11–24. 6186379 10.1016/0092-8674(82)90400-7

[78] Moll R, Divo M, Langbein L. The human keratins: biology and pathology. Histochem Cell Biol. 2008;129:705–33. 18461349 10.1007/s00418-008-0435-6 PMC2386534

[79] Salas PJ, Forteza R, Mashukova A. Multiple roles for keratin intermediate filaments in the regulation of epithelial barrier function and apico-basal polarity. Tissue Barriers. 2016;4:e1178368. 27583190 10.1080/21688370.2016.1178368 PMC4993576

[80] Giroux V, Stephan J, Chatterji P, Rhoades B, Wileyto EP, Klein-Szanto AJ, et al. Mouse Intestinal Krt15+ Crypt Cells Are Radio-Resistant and Tumor Initiating. Stem Cell Reports. 2018;10:1947–58. 29805107 10.1016/j.stemcr.2018.04.022 PMC5993649

[81] Morris RJ, Liu Y, Marles L, Yang Z, Trempus C, Li S, et al. Capturing and profiling adult hair follicle stem cells. Nat Biotechnol. 2004;22:411–7. 15024388 10.1038/nbt950

[82] Zhang Z, Wang H, Jin Y, Zhou J, Chu C, Tang F, et al. KRT15 in early breast cancer screening and correlation with HER2 positivity, pathological grade and N stage. Biomark Med. 2023;17:553–62. 37814985 10.2217/bmm-2023-0130

[83] Zhong P, Shu R, Wu H, Liu Z, Shen X, Hu Y. Low KRT15 expression is associated with poor prognosis in patients with breast invasive carcinoma. Exp Ther Med. 2021;21:305. 33717248 10.3892/etm.2021.9736 PMC7885068

[84] Rakha EA, Miligy IM, Gorringe KL, Toss MS, Green AR, Fox SB, et al. Invasion in breast lesions: the role of the epithelial-stroma barrier. Histopathology. 2018;72:1075–83. 29197112 10.1111/his.13446

[85] Englund JI, Bui H, Dinç DD, Paavolainen O, McKenna T, Laitinen S, et al. Laminin matrix adhesion regulates basal mammary epithelial cell identity. J Cell Sci. 2022. 10.1242/jcs.260232. 36468336 10.1242/jcs.260232

[86] Insua-Rodríguez J, Oskarsson T. The extracellular matrix in breast cancer. Adv Drug Deliv Rev. 2016;97:41–55. 26743193 10.1016/j.addr.2015.12.017

[87] Ghannam SF, Rutland CS, Allegrucci C, Mongan NP, Rakha E. Defining invasion in breast cancer: the role of basement membrane. J Clin Pathol. 2023;76:11–8. 36253088 10.1136/jcp-2022-208584

[88] Theocharis AD, Skandalis SS, Gialeli C, Karamanos NK. Extracellular matrix structure. Adv Drug Deliv Rev. 2016;97:4–27. 26562801 10.1016/j.addr.2015.11.001

[89] Lepucki A, Orlińska K, Mielczarek-Palacz A, Kabut J, Olczyk P, Komosińska-Vassev K. The role of extracellular matrix proteins in breast cancer. J Clin Med. 2022;11:1250. 35268340 10.3390/jcm11051250 PMC8911242

[90] Hanker AB, Estrada MV, Bianchini G, Moore PD, Zhao J, Cheng F, et al. Extracellular matrix/integrin signaling promotes resistance to combined inhibition of HER2 and PI3K in HER2^+^ breast cancer. Cancer Res. 2017;77:3280–92. 28396358 10.1158/0008-5472.CAN-16-2808 PMC5482178

[91] Shi W, Gerster K, Alajez NM, Tsang J, Waldron L, Pintilie M, et al. MicroRNA-301 mediates proliferation and invasion in human breast cancer. Cancer Res. 2011;71:2926–37. 21393507 10.1158/0008-5472.CAN-10-3369

[92] Wu CC, Chang SC, Zeng GY, Chu HW, Huang Y, Liu HP. Proteome analyses reveal positive association of COL2A1, MPO, TYMS, and IGFBP5 with canine mammary gland malignancy. Proteomics Clin Appl. 2019;13:e1800151. 30578659 10.1002/prca.201800151

[93] Pöschel A, Beebe E, Kunz L, Amini P, Guscetti F, Malbon A, et al. Identification of disease-promoting stromal components by comparative proteomic and transcriptomic profiling of canine mammary tumors using laser-capture microdissected FFPE tissue. Neoplasia. 2021;23:400–12. 33794398 10.1016/j.neo.2021.03.001 PMC8042244

[94] Peña L, Castaña M, Sanchez MA, Rodriguez A, Flores JM. Immunocytochemical study of type IV collagen and laminin in canine mammary tumours. Zentralbl Veterinarmed A. 1995;42:50–61. 8592880 10.1111/j.1439-0442.1995.tb00355.x

[95] Gaiko-Shcherbak A, Fabris G, Dreissen G, Merkel R, Hoffmann B, Noetzel E. The Acinar Cage: Basement Membranes Determine Molecule Exchange and Mechanical Stability of Human Breast Cell Acini. PLoS ONE. 2015;10: e0145174. 26674091 10.1371/journal.pone.0145174 PMC4684506

[96] Novaro V, Roskelley CD, Bissell MJ. Collagen-IV and laminin-1 regulate estrogen receptor alpha expression and function in mouse mammary epithelial cells. J Cell Sci. 2003;116(Pt 14):2975–86. 12808020 10.1242/jcs.00523 PMC2933217

[97] Wu Y, Ge G. Complexity of type IV collagens: from network assembly to function. Biol Chem. 2019;400:565–74. 30864416 10.1515/hsz-2018-0317

[98] Wu Y, Liu X, Zhu Y, Qiao Y, Gao Y, Chen J, Ge G. Type IV collagen α5 chain promotes luminal breast cancer progression through c-Myc-driven glycolysis. J Mol Cell Biol. 2023;14:mjac068. 36484686 10.1093/jmcb/mjac068 PMC10077331

[99] Hewitt RE, Powe DG, Morrell K, Balley E, Leach IH, Ellis IO, Turner DR. Laminin and collagen IV subunit distribution in normal and neoplastic tissues of colorectum and breast. Br J Cancer. 1997;75:221–9. 9010030 10.1038/bjc.1997.37 PMC2063263

[100] Nakano S, Iyama K, Ogawa M, Yoshioka H, Sado Y, Oohashi T, Ninomiya Y. Differential tissular expression and localization of type IV collagen alpha1(IV), alpha2(IV), alpha5(IV), and alpha6(IV) chains and their mRNA in normal breast and in benign and malignant breast tumors. Lab Invest. 1999;79:281–92. 10092064

[101] Tao D, Wang Y, Zhang X, Wang C, Yang D, Chen J, Long Y, Jiang Y, Zhou X, Zhang N. Identification of angiogenesis-related prognostic biomarkers associated with immune cell infiltration in breast cancer. Front Cell Dev Biol. 2022;10:853324. 35602610 10.3389/fcell.2022.853324 PMC9121305

[102] Miller KA, Chung J, Lo D, Jones JC, Thimmapaya B, Weitzman SA. Inhibition of laminin-5 production in breast epithelial cells by overexpression of p300. J Biol Chem. 2000;275:8176–82. 10713141 10.1074/jbc.275.11.8176

[103] Henning K, Berndt A, Katenkamp D, Kosmehl H. Loss of laminin-5 in the epithelium-stroma interface: an immunohistochemical marker of malignancy in epithelial lesions of the breast. Histopathology. 1999;34:305–9. 10231397 10.1046/j.1365-2559.1999.00634.x

[104] Sathyanarayana UG, Padar A, Huang CX, Suzuki M, Shigematsu H, Bekele BN, Gazdar AF. Aberrant promoter methylation and silencing of laminin-5-encoding genes in breast carcinoma. Clin Cancer Res. 2003;9:6389–94. 14695139

[105] Luo R, Chong W, Wei Q, Zhang Z, Wang C, Ye Z, Abu-Khalaf MM, Silver DP, Stapp RT, Jiang W, Myers RE, Li B, Cristofanilli M, Yang H. Whole-exome sequencing identifies somatic mutations and intratumor heterogeneity in inflammatory breast cancer. NPJ Breast Cancer. 2021;7:72. 34075047 10.1038/s41523-021-00278-w PMC8169683

[106] Jansson M, Billing O, Herdenberg C, Lundin C, Tolockiene E, Nazemroaya A, Sund M. Expression and circulating levels of perlecan in breast cancer - implications for oestrogen dependent stromal remodeling. J Mammary Gland Biol Neoplasia. 2020;25:69–77. 32124140 10.1007/s10911-020-09447-2

[107] Ishihara A, Yoshida T, Tamaki H, Sakakura T. Tenascin expression in cancer cells and stroma of human breast cancer and its prognostic significance. Clin Cancer Res. 1995;1:1035–41. 9816077

[108] Tsunoda T, Inada H, Kalembeyi I, Imanaka-Yoshida K, Sakakibara M, Okada R, et al. Involvement of large tenascin-c splice variants in breast cancer progression. Am J Pathol. 2003;162:1857–67. 12759243 10.1016/S0002-9440(10)64320-9 PMC1868127

[109] Faustino AM, van Garderen E, Schalken JA, Nederbragt H. Tenascin expression in normal, hyperplastic, dysplastic and neoplastic canine mammary tissues. J Comp Pathol. 2002;126:1–8. 11814316 10.1053/jcpa.2001.0519

[110] Yoshimura H, Michishita M, Ohkusu-Tsukada K, Takahashi K. Increased presence of stromal myofibroblasts and tenascin-C with malignant progression in canine mammary tumors. Vet Pathol. 2011;48:313–21. 20571146 10.1177/0300985810369901

[111] Yoshimura H, Michishita M, Ohkusu-Tsukada K, Matsuda Y, Ishiwata T, Naito Z, et al. Cellular sources of tenascin-C in canine mammary carcinomas. Vet Pathol. 2015;52:92–6. 24565830 10.1177/0300985814522817

[112] Oskarsson T, Acharyya S, Zhang XH, Vanharanta S, Tavazoie SF, Morris PG, et al. Breast cancer cells produce tenascin C as a metastatic niche component to colonize the lungs. Nat Med. 2011;17:867–74. 21706029 10.1038/nm.2379 PMC4020577

[113] Murdamoothoo D, Sun Z, Yilmaz A, Riegel G, Abou-Faycal C, Deligne C, et al. Tenascin-C immobilizes infiltrating T lymphocytes through CXCL12 promoting breast cancer progression. EMBO Mol Med. 2021;13(6): e13270. 33988305 10.15252/emmm.202013270 PMC8185552

[114] Rahimmanesh I, Fatehi R, Khanahmad H. Identification of significant genes and pathways associated with tenascin-C in cancer progression by bioinformatics analysis. Adv Biomed Res. 2022;11:17. 35386538 10.4103/abr.abr_201_20 PMC8977614

[115] Adams JC, Lawler J. The thrombospondins. Cold Spring Harb Perspect Biol. 2011;3: a009712. 21875984 10.1101/cshperspect.a009712 PMC3179333

[116] Yee KO, Connolly CM, Duquette M, Kazerounian S, Washington R, Lawler J. The effect of thrombospondin-1 on breast cancer metastasis. Breast Cancer Res Treat. 2009;114:85–96. 18409060 10.1007/s10549-008-9992-6 PMC2631620

[117] Albo D, Berger DH, Wang TN, Hu X, Rothman V, Tuszynski GP. Thrombospondin-1 and transforming growth factor-beta l promote breast tumor cell invasion through up-regulation of the plasminogen/plasmin system. Surgery. 1997;122:493–9 discussion 499–500. 9288157 10.1016/s0039-6060(97)90043-x

[118] Martin-Manso G, Calzada MJ, Chuman Y, Sipes JM, Xavier CP, Wolf V, et al. sFRP-1 binds via its netrin-related motif to the N-module of thrombospondin-1 and blocks thrombospondin-1 stimulation of MDA-MB-231 breast carcinoma cell adhesion and migration. Arch Biochem Biophys. 2011;509:147–56. 21402050 10.1016/j.abb.2011.03.004 PMC3085965

[119] Sun S, Dong H, Yan T, Li J, Liu B, Shao P, et al. Role of TSP-1 as prognostic marker in various cancers: a systematic review and meta-analysis. BMC Med Genet. 2020;21:139. 32600280 10.1186/s12881-020-01073-3 PMC7325168

[120] Padmanaban V, Krol I, Suhail Y, Szczerba BM, Aceto N, Bader JS, et al. E-cadherin is required for metastasis in multiple models of breast cancer. Nature. 2019;573:439–44. 31485072 10.1038/s41586-019-1526-3 PMC7365572

[121] Burandt E, Lübbersmeyer F, Gorbokon N, Büscheck F, Luebke AM, Menz A, et al. E-Cadherin expression in human tumors: a tissue microarray study on 10,851 tumors. Biomark Res. 2021;9:44. 34090526 10.1186/s40364-021-00299-4 PMC8180156

[122] Tanoue T, Nishida E. Molecular recognitions in the MAP kinase cascades. Cell Signal. 2003;15:455–62. 12639708 10.1016/s0898-6568(02)00112-2

[123] Plotnikov A, Zehorai E, Procaccia S, Seger R. The MAPK cascades: signaling components, nuclear roles and mechanisms of nuclear translocation. Biochim Biophys Acta. 2011;1813:1619–33. 21167873 10.1016/j.bbamcr.2010.12.012

[124] Pham TT, Angus SP, Johnson GL. MAP3K1: genomic alterations in cancer and function in promoting cell survival or apoptosis. Genes Cancer. 2013;4:419–26. 24386504 10.1177/1947601913513950 PMC3877667

[125] Zhang Z, Yamashita H, Toyama T, Sugiura H, Ando Y, Mita K, et al. NCOR1 mRNA is an independent prognostic factor for breast cancer. Cancer Lett. 2006;237:123–9. 16019133 10.1016/j.canlet.2005.05.046

[126] Aref-Eshghi E, Lin F, Li MM, Zhong Y. The oncogenic roles of NTRK fusions and methods of molecular diagnosis. Cancer Genet. 2021;258–259:110–9. 34710798 10.1016/j.cancergen.2021.10.005

[127] Stephens P, Edkins S, Davies H, Greenman C, Cox C, Hunter C, et al. A screen of the complete protein kinase gene family identifies diverse patterns of somatic mutations in human breast cancer. Nat Genet. 2005;37:590–2. 15908952 10.1038/ng1571

[128] Medford AJ, Oshry L, Boyraz B, Kiedrowski L, Menshikova S, Butusova A, et al. TRK inhibitor in a patient with metastatic triple-negative breast cancer and *NTRK* fusions identified *via* cell-free DNA analysis. Ther Adv Med Oncol. 2023. 10.1177/17588359231152844. 36743521 10.1177/17588359231152844 PMC9893401

[129] Zito Marino F, Buono S, Montella M, Giannatiempo R, Messina F, Casaretta G, et al. NTRK gene aberrations in triple-negative breast cancer: detection challenges using IHC, FISH, RT-PCR, and NGS. J Pathol Clin Res. 2023;9:367–77. 37143440 10.1002/cjp2.324 PMC10397374

[130] Ressel L, Puleio R, Loria GR, Vannozzi I, Millanta F, Caracappa S, et al. HER-2 expression in canine morphologically normal, hyperplastic and neoplastic mammary tissues and its correlation with the clinical outcome. Res Vet Sci. 2013;94:299–305. 23141215 10.1016/j.rvsc.2012.09.016

[131] Burrai GP, Tanca A, De Miglio MR, Abbondio M, Pisanu S, Polinas M, et al. Investigation of HER2 expression in canine mammary tumors by antibody-based, transcriptomic and mass spectrometry analysis: is the dog a suitable animal model for human breast cancer? Tumour Biol. 2015;36:9083–91. 26088453 10.1007/s13277-015-3661-2

[132] Varallo GR, Gelaleti GB, Maschio-Signorini LB, Moschetta MG, Lopes JR, De Nardi AB, et al. Prognostic phenotypic classification for canine mammary tumors. Oncol Lett. 2019;18:6545–53. 31807173 10.3892/ol.2019.11052 PMC6876320

[133] Pastor N, Ezquerra LJ, Santella M, Caballé NC, Tarazona R, Durán ME. Prognostic significance of immunohistochemical markers and histological classification in malignant canine mammary tumours. Vet Comp Oncol. 2020;18:753–62. 32336005 10.1111/vco.12603 PMC7754150

[134] Seung BJ, Cho SH, Kim SH, Lim HY, Sur JH. Quantitative analysis of HER2 mRNA expression by RNA in situ hybridization in canine mammary gland tumors: Comparison with immunohistochemistry analysis. PLoS ONE. 2020;15: e0229031. 32059046 10.1371/journal.pone.0229031 PMC7021316

[135] Bergholtz H, Lien T, Lingaas F, Sørlie T. Comparative analysis of the molecular subtype landscape in canine and human mammary gland tumors. J Mammary Gland Biol Neoplasia. 2022;27:171–83. 35932380 10.1007/s10911-022-09523-9 PMC9433360

[136] Hsu WL, Huang HM, Liao JW, Wong ML, Chang SC. Increased survival in dogs with malignant mammary tumours overexpressing HER-2 protein and detection of a silent single nucleotide polymorphism in the canine HER-2 gene. Vet J. 2009;180:116–23. 18061495 10.1016/j.tvjl.2007.10.013

[137] Canadas-Sousa A, Santos M, Medeiros R, Dias-Pereira P. Single nucleotide polymorphisms influence histological type and grade of canine malignant mammary tumours. J Comp Pathol. 2019;172:72–9. 31690419 10.1016/j.jcpa.2019.08.010

[138] Borge KS, Nord S, Van Loo P, Lingjærde OC, Gunnes G, Alnæs GI, et al. Canine mammary tumours are affected by frequent copy number aberrations, including amplification of MYC and Loss of PTEN. PLoS ONE. 2015;10:e0126371. 25955013 10.1371/journal.pone.0126371 PMC4425491

[139] Arendt ML, Sakthikumar S, Melin M, Elvers I, Rivera P, Larsen M, et al. PIK3CA is recurrently mutated in canine mammary tumors, similarly to in human mammary neoplasia. Sci Rep. 2023;13:632. 36635367 10.1038/s41598-023-27664-7 PMC9837039

[140] Kim SH, Seung BJ, Cho SH, Lim HY, Bae MK, Sur JH. Dysregulation of PI3K/Akt/PTEN pathway in canine mammary tumor. Animals (Basel). 2021;11:2079. 34359206 10.3390/ani11072079 PMC8300234

[141] Reinhardt K, Stückrath K, Hartung C, Kaufhold S, Uleer C, Hanf V, et al. PIK3CA-mutations in breast cancer. Breast Cancer Res Treat. 2022;196:483–93. 36279023 10.1007/s10549-022-06637-w PMC9633529

[142] Li Y, Zhang SW, Xie MY, Zhang T. PhenoDriver: interpretable framework for studying personalized phenotype-associated driver genes in breast cancer. Brief Bioinform. 2023. 10.1093/bib/bbad291. 37738403 10.1093/bib/bbad291

[143] Deb S, Chakrabarti A, Fox SB. Prognostic and predictive biomarkers in familial breast cancer. Cancers (Basel). 2023;15:1346. 36831687 10.3390/cancers15041346 PMC9953970

[144] Jin J, Cao J, Li B, Li T, Zhang J, Cao J, et al. Landscape of DNA damage response gene alterations in breast cancer: A comprehensive investigation. Cancer. 2023;129:845–59. 36655350 10.1002/cncr.34618

[145] Nieto A, Pérez-Alenza MD, Del Castillo N, Tabanera E, Castaño M, Peña L. BRCA1 expression in canine mammary dysplasias and tumours: relationship with prognostic variables. J Comp Pathol. 2003;128:260–8. 12834609 10.1053/jcpa.2002.0631

[146] Rivera P, Melin M, Biagi T, Fall T, Häggström J, Lindblad-Toh K, et al. Mammary tumor development in dogs is associated with BRCA1 and BRCA2. Cancer Res. 2009;69:8770–4. 19887619 10.1158/0008-5472.CAN-09-1725

[147] Borge KS, Børresen-Dale AL, Lingaas F. Identification of genetic variation in 11 candidate genes of canine mammary tumour. Vet Comp Oncol. 2011;9:241–50. 22077404 10.1111/j.1476-5829.2010.00250.x

[148] Im KS, Kim IH, Kim NH, Lim HY, Kim JH, Sur JH. Breed-related differences in altered BRCA1 expression, phenotype and subtype in malignant canine mammary tumors. Vet J. 2013;195:366–72. 22901454 10.1016/j.tvjl.2012.07.014

[149] Enginler SO, Akış I, Toydemir TS, Oztabak K, Haktanir D, Gündüz MC, et al. Genetic variations of BRCA1 and BRCA2 genes in dogs with mammary tumours. Vet Res Commun. 2014;38:21–7. 24122022 10.1007/s11259-013-9577-7

[150] Qiu HB, Sun WD, Yang X, Jiang QY, Chen S, Lin DG. Promoter mutation and reduced expression of BRCA1 in canine mammary tumors. Res Vet Sci. 2015;103:143–8. 26679809 10.1016/j.rvsc.2015.10.003

[151] Sun W, Yang X, Qiu H, Zhang D, Wang H, Huang J, et al. Relationship between three novel SNPs of BRCA1 and canine mammary tumors. J Vet Med Sci. 2015;77:1541–3. 26156012 10.1292/jvms.15-0044 PMC4667680

[152] Qiu H, Lin D. Roles of DNA mutation in the coding region and DNA methylation in the 5’ flanking region of BRCA1 in canine mammary tumors. J Vet Med Sci. 2016;78:943–9. 26888582 10.1292/jvms.15-0557 PMC4937153

[153] Di Giacomo D, Di Domenico M, Defourny SVP, Malatesta D, Di Teodoro G, Martino M, et al. Validation of ampliSeq NGS Panel for *BRCA1* and *BRCA2* variant detection in canine formalin-fixed paraffin-embedded mammary tumors. Life (Basel). 2022;12:851. 35743882 10.3390/life12060851 PMC9225004

[154] Conciatori F, Ciuffreda L, Bazzichetto C, Falcone I, Pilotto S, Bria E, et al. mTOR cross-talk in cancer and potential for combination therapy. Cancers (Basel). 2018;10:23. 29351204 10.3390/cancers10010023 PMC5789373

[155] Sato T, Nakashima A, Guo L, Coffman K, Tamanoi F. Single amino-acid changes that confer constitutive activation of mTOR are discovered in human cancer. Oncogene. 2010;29:2746–52. 20190810 10.1038/onc.2010.28 PMC2953941

[156] Hardt M, Chantaravisoot N, Tamanoi F. Activating mutations of TOR (target of rapamycin). Genes Cells. 2011;16:141–51. 21210909 10.1111/j.1365-2443.2010.01482.x PMC3116645

[157] Grabiner BC, Nardi V, Birsoy K, Possemato R, Shen K, Sinha S, et al. A diverse array of cancer-associated MTOR mutations are hyperactivating and can predict rapamycin sensitivity. Cancer Discov. 2014;4:554–63. 24631838 10.1158/2159-8290.CD-13-0929 PMC4012430

[158] Walsh S, Flanagan L, Quinn C, Evoy D, McDermott EW, Pierce A, et al. mTOR in breast cancer: differential expression in triple-negative and non-triple-negative tumors. Breast. 2012;21:178–82. 21963359 10.1016/j.breast.2011.09.008

[159] Delgado L, Gärtner F, Dias PP. Activation of Mammalian target of rapamycin in canine mammary carcinomas: an immunohistochemical study. J Comp Pathol. 2015;152:138–44. 25670666 10.1016/j.jcpa.2014.12.004

[160] Asproni P, Millanta F, Ressel L, Podestà F, Parisi F, Vannozzi I, et al. An immunohistochemical study of the PTEN/AKT pathway involvement in canine and feline mammary tumors. Animals. 2021;11:365. 33535663 10.3390/ani11020365 PMC7912927

[161] Perossi IFS, Saito MM, Varallo GR, de Godoy BLV, Colombo J, Zuccari DAPC. Protein expression of PI3K/AKT/mTOR pathway targets validated by gene expression and its correlation with prognosis in canine mammary cancer. J Mammary Gland Biol Neoplasia. 2022;27:241–52. 36323932 10.1007/s10911-022-09527-5

[162] Xu J, Chen Y, Olopade OI. MYC and breast cancer. Genes Cancer. 2010;1:629–40. 21779462 10.1177/1947601910378691 PMC3092228

[163] Budurlean L, Baker M, Broach J. Rare MYC-N11S germline mutation indicative of inherited breast cancer in a multigeneration family. BMJ Case Rep. 2022. 10.1136/bcr-2022-251336. 36368728 10.1136/bcr-2022-251336 PMC9660511

[164] Jeong EG, Kim MS, Nam HK, Min CK, Lee S, Chung YJ, et al. Somatic mutations of JAK1 and JAK3 in acute leukemias and solid cancers. Clin Cancer Res. 2008;14:3716–21. 18559588 10.1158/1078-0432.CCR-07-4839

[165] Liu F, Wu H. Identification of prognostic biomarkers and molecular targets among JAK family in breast cancer. J Inflamm Res. 2021;14:97–114. 33469338 10.2147/JIR.S284889 PMC7813467

[166] Nassar A, Zekri ARN, Elberry MH, Lymona AM, Lotfy MM, Abouelhoda M, et al. Somatic mutations alter interleukin signaling pathways in grade II invasive breast cancer patients: an Egyptian experience. Curr Issues Mol Biol. 2022;44:5890–901. 36547062 10.3390/cimb44120401 PMC9777163

[167] Eum SY, Lee YW, Hennig B, Toborek M. Interplay between epidermal growth factor receptor and Janus kinase 3 regulates polychlorinated biphenyl-induced matrix metalloproteinase-3 expression and transendothelial migration of tumor cells. Mol Cancer Res. 2006;4:361–70. 16778083 10.1158/1541-7786.MCR-05-0119

[168] Ye Q, Kantonen S, Gomez-Cambronero J. Serum deprivation confers the MDA-MB-231 breast cancer line with an EGFR/JAK3/PLD2 system that maximizes cancer cell invasion. J Mol Biol. 2013;425:755–66. 23238254 10.1016/j.jmb.2012.11.035 PMC3568238

[169] Liu X, Wei T, Gao ZD, Zhao XL, Wu HQ. [Janus kinase 3 facilitates the migration of breast cancer cells by store-operated calcium channel]. Yan J Sheng Li Xue Bao. 2019;71:874–82. 31879743

[170] Fallahian F, Karami-Tehrani F, Salami S, Aghaei M. Cyclic GMP induced apoptosis via protein kinase G in oestrogen receptor-positive and -negative breast cancer cell lines. FEBS J. 2011;278:3360–9. 21777390 10.1111/j.1742-4658.2011.08260.x

[171] Fallahian F, Karami-Tehrani F, Salami S. Induction of apoptosis by type Iβ protein kinase G in the human breast cancer cell lines MCF-7 and MDA-MB-468. Cell Biochem Funct. 2012;30:183–90. 22095901 10.1002/cbf.1831

[172] Karami-Tehrani F, Fallahian F, Atri M. Expression of cGMP-dependent protein kinase, PKGIα, PKGIβ, and PKGII in malignant and benign breast tumors. Tumour Biol. 2012;33:1927–32. 22791569 10.1007/s13277-012-0453-9

[173] Bennett L, Mallon EA, Horgan PG, Paul A, McMillan DC, Edwards J. The relationship between members of the canonical NF-κB pathway, components of tumour microenvironment and survival in patients with invasive ductal breast cancer. Oncotarget. 2017;8:33002–13. 28423692 10.18632/oncotarget.16031 PMC5464845

[174] Biswas DK, Shi Q, Baily S, Strickland I, Ghosh S, Pardee AB, et al. NF-kappa B activation in human breast cancer specimens and its role in cell proliferation and apoptosis. Proc Natl Acad Sci U S A. 2004;101:10137–42. 15220474 10.1073/pnas.0403621101 PMC454178

[175] Hou J, Ye X, Wang Y, Li C. Stratification of estrogen receptor-negative breast cancer patients by integrating the somatic mutations and transcriptomic data. Front Genet. 2021;12;610087. 33613637 10.3389/fgene.2021.610087 PMC7886807

[176] Vogelstein B, Papadopoulos N, Velculescu VE, Zhou S, Diaz LA Jr, Kinzler KW. Cancer genome landscapes. Science. 2013;339:1546–58. 23539594 10.1126/science.1235122 PMC3749880

[177] Du XW, Li G, Liu J, Zhang CY, Liu Q, Wang H, Chen TS. Comprehensive analysis of the cancer driver genes in breast cancer demonstrates their roles in cancer prognosis and tumor microenvironment. World J Surg Oncol. 2021;19:273. 34507558 10.1186/s12957-021-02387-z PMC8434726

[178] Srinivasan K, Strickland P, Valdes A, Shin GC, Hinck L. Netrin-1/neogenin interaction stabilizes multipotent progenitor cap cells during mammary gland morphogenesis. Dev Cell. 2003;4:371–82. 12636918 10.1016/s1534-5807(03)00054-6

[179] Lee JE, Kim HJ, Bae JY, Kim SW, Park JS, Shin HJ, et al. Neogenin expression may be inversely correlated to the tumorigenicity of human breast cancer. BMC Cancer. 2005;5:154. 16324219 10.1186/1471-2407-5-154 PMC1322231

[180] Villanueva AA, Puvogel S, Lois P, Muñoz-Palma E, Ramírez Orellana M, Lubieniecki F, et al. The Netrin-4/Laminin γ1/Neogenin-1 complex mediates migration in SK-N-SH neuroblastoma cells. Cell Adh Migr. 2019;13:33–40. 30160193 10.1080/19336918.2018.1506652 PMC6527380

[181] Zhang M, Zhou Z, Pan XK, Zhou YJ, Li HO, Qiu PS, et al. Identification of NEO1 as a prognostic biomarker and its effects on the progression of colorectal cancer. Cancer Cell Int. 2020;20:510. 33088218 10.1186/s12935-020-01604-1 PMC7568410

[182] Li G, Huang J, Chen S, He Y, Wang Z, Peng J. High expression of ATP6V1C2 predicts unfavorable overall survival in patients with colon adenocarcinoma. Front Genet. 2022;13:930876. 36212133 10.3389/fgene.2022.930876 PMC9532742

[183] McConnell M, Feng S, Chen W, Zhu G, Shen D, Ponnazhagan S, et al. Osteoclast proton pump regulator Atp6v1c1 enhances breast cancer growth by activating the mTORC1 pathway and bone metastasis by increasing V-ATPase activity. Oncotarget. 2017;8:47675–90. 28504970 10.18632/oncotarget.17544 PMC5564597

[184] Brackertz M, Boeke J, Zhang R, Renkawitz R. Two highly related p66 proteins comprise a new family of potent transcriptional repressors interacting with MBD2 and MBD3. J Biol Chem. 2002;277:40958–66. 12183469 10.1074/jbc.M207467200

[185] Brackertz M, Gong Z, Leers J, Renkawitz R. p66alpha and p66beta of the Mi-2/NuRD complex mediate MBD2 and histone interaction. Nucleic Acids Res. 2006;34:397–406. 16415179 10.1093/nar/gkj437 PMC1331983

[186] Lai AY, Wade PA. Cancer biology and NuRD: a multifaceted chromatin remodelling complex. Nat Rev Cancer. 2011;11:588–96. 21734722 10.1038/nrc3091 PMC4157524

[187] Torchy MP, Hamiche A, Klaholz BP. Structure and function insights into the NuRD chromatin remodeling complex. Cell Mol Life Sci. 2015;72:2491–507. 25796366 10.1007/s00018-015-1880-8 PMC11114056

[188] Grzeskowiak CL, Kundu ST, Mo X, Ivanov AA, Zagorodna O, Lu H, et al. In vivo screening identifies GATAD2B as a metastasis driver in KRAS-driven lung cancer. Nat Commun. 2018;9:2732. 30013058 10.1038/s41467-018-04572-3 PMC6048166

[189] Fang Y, Zhong Q, Wang Y, Gu C, Liu S, Li A, et al. CPEB3 functions as a tumor suppressor in colorectal cancer via JAK/STAT signaling. Aging (Albany NY). 2020;12:21404–22. 33146632 10.18632/aging.103893 PMC7695424

[190] Liang ZZ, Zhang YX, Zhu RM, Li YL, Jiang HM, Li RB, et al. Identification of epigenetic modifications mediating the antagonistic effect of selenium against cadmium-induced breast carcinogenesis. Environ Sci Pollut Res Int. 2022;29:22056–68. 34773240 10.1007/s11356-021-17355-z

[191] Zhong Q, Fang Y, Lai Q, Wang S, He C, Li A, et al. CPEB3 inhibits epithelial-mesenchymal transition by disrupting the crosstalk between colorectal cancer cells and tumor-associated macrophages via IL-6R/STAT3 signaling. J Exp Clin Cancer Res. 2020;39:132. 32653013 10.1186/s13046-020-01637-4 PMC7353816

[192] Wang H, Wang C, Peng G, Yu D, Cui XG, Sun YH, et al. Capping protein regulator and myosin 1 linker 3 is required for tumor metastasis. Mol Cancer Res. 2020;18:240–52. 31694931 10.1158/1541-7786.MCR-19-0722

[193] Wang CA, Drasin D, Pham C, Jedlicka P, Zaberezhnyy V, Guney M, et al. Homeoprotein Six2 promotes breast cancer metastasis via transcriptional and epigenetic control of E-cadherin expression. Cancer Res. 2014;74:7357–70. 25348955 10.1158/0008-5472.CAN-14-0666 PMC4268359

[194] Shriver M, Stroka KM, Vitolo MI, Martin S, Huso DL, Konstantopoulos K, et al. Loss of giant obscurins from breast epithelium promotes epithelial-to-mesenchymal transition, tumorigenicity and metastasis. Oncogene. 2015;34:4248–59. 25381817 10.1038/onc.2014.358 PMC4426246

[195] Rajendran BK, Deng CX. A comprehensive genomic meta-analysis identifies confirmatory role of *OBSCN* gene in breast tumorigenesis. Oncotarget. 2017;8:102263–76. 29254242 10.18632/oncotarget.20404 PMC5731952

[196] Rajendran BK, Deng CX. Characterization of potential driver mutations involved in human breast cancer by computational approaches. Oncotarget. 2017;8:50252–72. 28477017 10.18632/oncotarget.17225 PMC5564847

[197] Bing Z, Tian J, Zhang J, Li X, Wang X, Yang K. An integrative model of miRNA and mRNA expression signature for patients of breast invasive carcinoma with radiotherapy prognosis. Cancer Biother Radiopharm. 2016;31:253–60. 27610468 10.1089/cbr.2016.2059

[198] Chiarella E, Aloisio A, Scicchitano S, Bond HM, Mesuraca M. Regulatory role of microRNAs targeting the transcription co-factor ZNF521 in normal tissues and cancers. Int J Mol Sci. 2021;22:8461. 34445164 10.3390/ijms22168461 PMC8395128

[199] Jeffries KA, Dempsey DR, Farrell EK, Anderson RL, Garbade GJ, Gurina TS, et al. Glycine N-acyltransferase-like 3 is responsible for long-chain N-acylglycine formation in N18TG2 cells. J Lipid Res. 2016;57:781–90. 27016726 10.1194/jlr.M062042 PMC4847626

[200] Uehiro N, Sato F, Pu F, Tanaka S, Kawashima M, Kawaguchi K, et al. Circulating cell-free DNA-based epigenetic assay can detect early breast cancer. Breast Cancer Res. 2016;18:129. 27993161 10.1186/s13058-016-0788-z PMC5168705

[201] Veldhoen N, Watterson J, Brash M, Milner J. Identification of tumour-associated and germ line p53 mutations in canine mammary cancer. Br J Cancer. 1999;81:409–15. 10507764 10.1038/sj.bjc.6690709 PMC2362910

[202] Lee CH, Kweon OK. Mutations of p53 tumor suppressor gene in spontaneous canine mammary tumors. J Vet Sci. 2002;3:321–5. 12819382

[203] Abe Y, Matsumoto S, Wei S, Nezu K, Miyoshi A, Kito K, et al. Cloning and characterization of a p53-related protein kinase expressed in interleukin-2-activated cytotoxic T-cells, epithelial tumor cell lines, and the testes. J Biol Chem. 2001;276:44003–11. 11546806 10.1074/jbc.M105669200

[204] Capra M, Nuciforo PG, Confalonieri S, Quarto M, Bianchi M, Nebuloni M, et al. Frequent alterations in the expression of serine/threonine kinases in human cancers. Cancer Res. 2006;66:8147–54. 16912193 10.1158/0008-5472.CAN-05-3489

[205] Mirza-Aghazadeh-Attari M, Mohammadzadeh A, Yousefi B, Mihanfar A, Karimian A, Majidinia M. 53BP1: A key player of DNA damage response with critical functions in cancer. DNA Repair (Amst). 2019;73:110–9. 30497961 10.1016/j.dnarep.2018.11.008

[206] Gerlinger M, Rowan AJ, Horswell S, Math M, Larkin J, Endesfelder D, et al. Intratumor heterogeneity and branched evolution revealed by multiregion sequencing. N Engl J Med. 2012;366:883–92. 22397650 10.1056/NEJMoa1113205 PMC4878653

[207] Hernandez L, Wilkerson PM, Lambros MB, Campion-Flora A, Rodrigues DN, Gauthier A, et al. Genomic and mutational profiling of ductal carcinomas in situ and matched adjacent invasive breast cancers reveals intra-tumour genetic heterogeneity and clonal selection. J Pathol. 2012;227:42–52. 22252965 10.1002/path.3990 PMC4975517

[208] Seferbekova Z, Lomakin A, Yates LR, Gerstung M. Spatial biology of cancer evolution. Nat Rev Genet. 2023;24:295–313. 36494509 10.1038/s41576-022-00553-x

