# Supplementary material for: Differential somatic coding variant landscapes between laser microdissected luminal epithelial cells from canine mammary invasive ductal solid carcinoma and comedocarcinoma
Source: BMC Cancer. 2024 Dec 18;24:1524. doi: 10.1186/s12885-024-13239-w (PMC11657561; doi:10.1186/s12885-024-13239-w)
Supplement: Supplementary file 2 — Additional file 2. Figure S1 Preseq2 complexity estimation and yield prediction for PicoPLEX libraries. Black line in A–C represents standard library, and colored lines low-coverage libraries sequenced at 1x. A Normal lobular. B Solid carcinoma. C Comedocarcinoma. Both normal sample libraries (IDs DOG_MAMMA_28 and DOG_MAMMA_32), one solid carcinoma sample library (ID DOG_MAMMA_1), and one comedocarcinoma sample library (ID DOG_MAMMA_10) were selected for further sequencing. [file 12885_2024_13239_MOESM2_ESM.pdf]

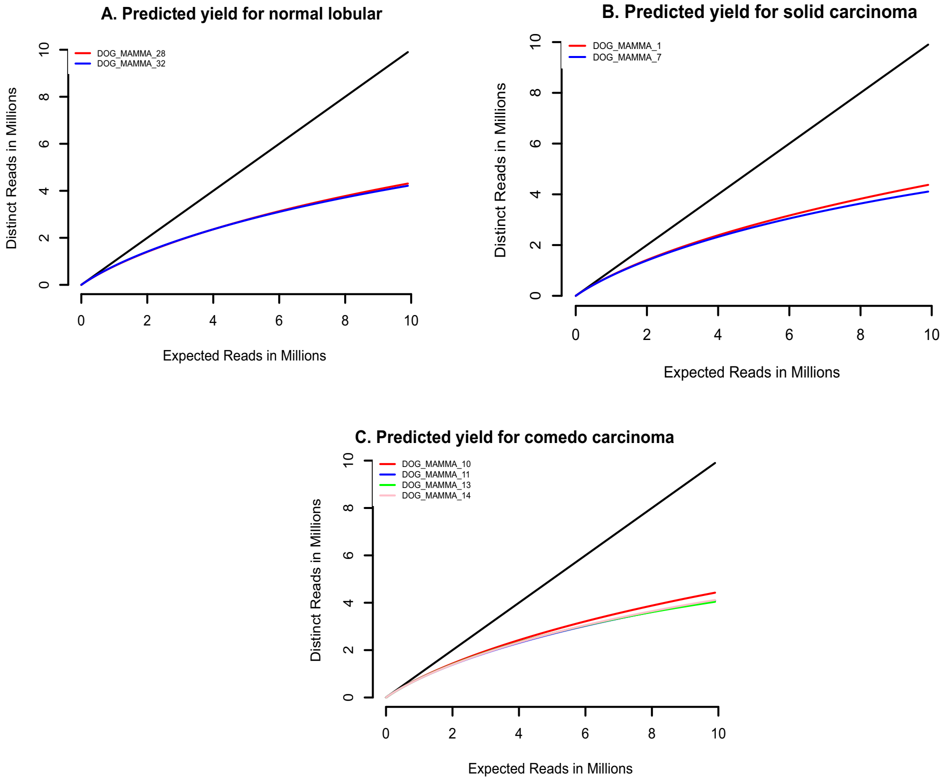
A. Predicted yield for normal lobular
DOG_MAMMA_28
DOG_MAMMA_32
Distinct Reads in Millions
Expected Reads in Millions
0
2
4
6
8
10
B. Predicted yield for solid carcinoma
DOG_MAMMA_1
DOG_MAMMA_7
Distinct Reads in Millions
Expected Reads in Millions
C. Predicted yield for comedo carcinoma
DOG_MAMMA_10
DOG_MAMMA_11
DOG_MAMMA_13
DOG_MAMMA_14
Distinct Reads in Millions
Expected Reads in Millions
