# Supplementary figures and images for: Differential somatic coding variant landscapes between laser microdissected luminal epithelial cells from canine mammary invasive ductal solid carcinoma and comedocarcinoma
Source: BMC Cancer. 2024 Dec 18;24:1524. doi: 10.1186/s12885-024-13239-w (PMC11657561; doi:10.1186/s12885-024-13239-w)

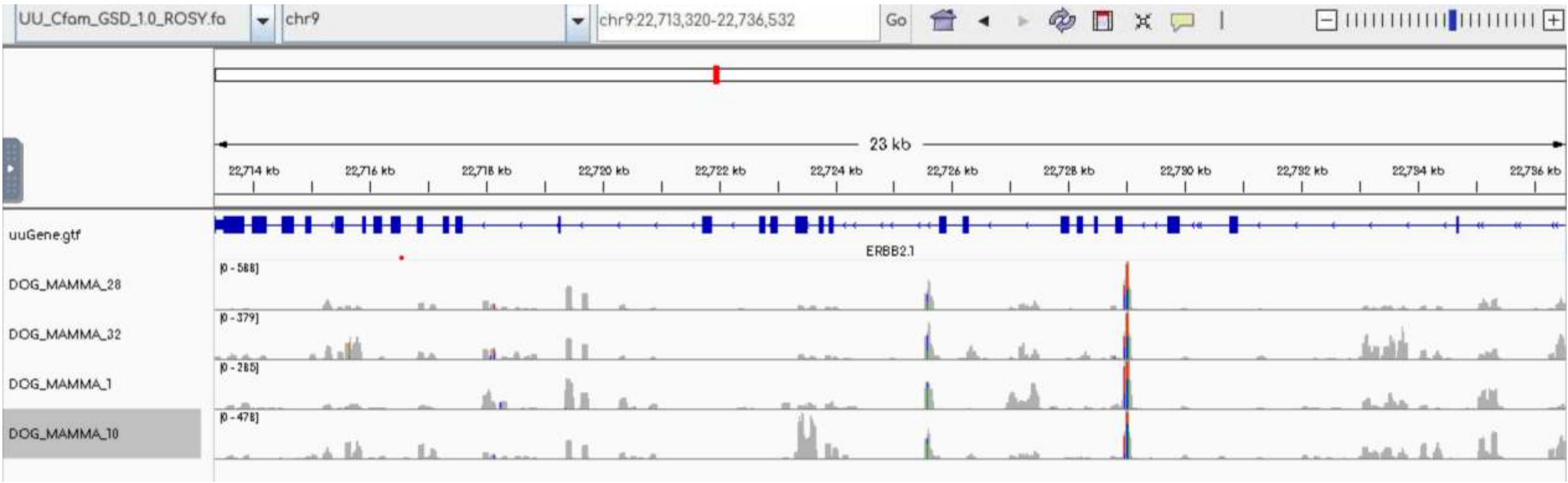

Supplement: Supplementary file 3 — Additional file 3. Figure S2 IGV (Integrative Genome Viewer) coverage plot for gene ERBB2 (NM_001003217) and region (chr9:22,713,320–22,736,532). An increase in coverage of reads from the BAM file is observed in solid carcinoma (ID DOG_MAMMA_1) and comedocarcinoma (ID DOG_MAMMA_10) samples compared to the two normal samples (IDs DOG_MAMMA_28 and DOG_MAMMA_32) in regions chr9:22,723,000–22,725,00. [file 12885_2024_13239_MOESM3_ESM.pdf]

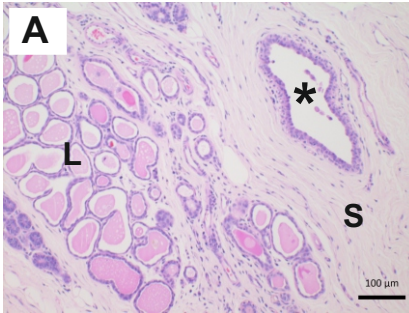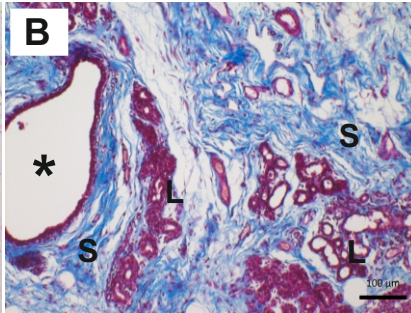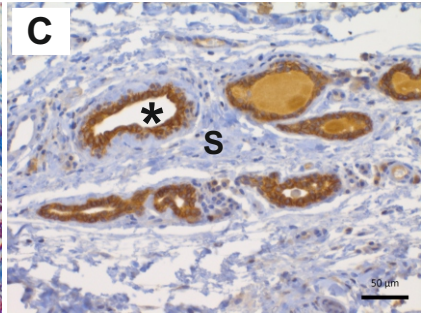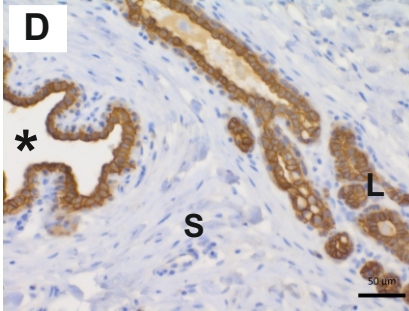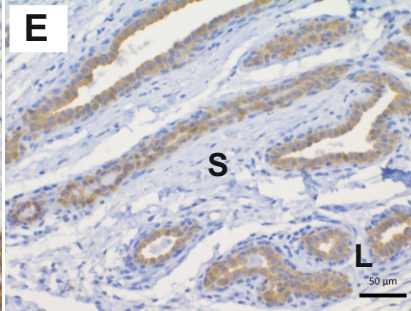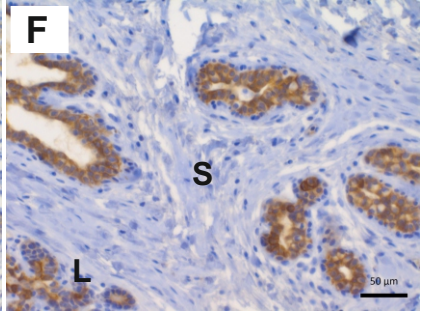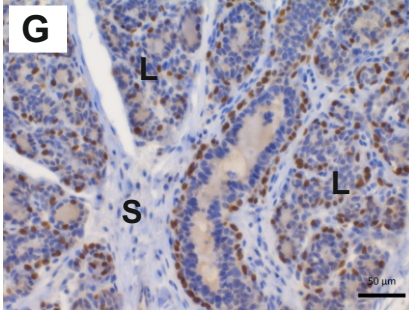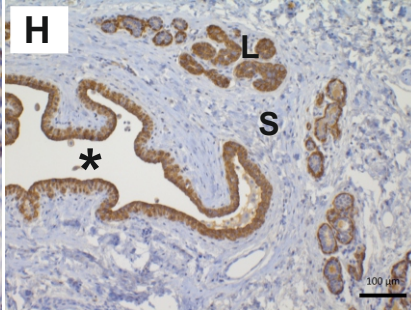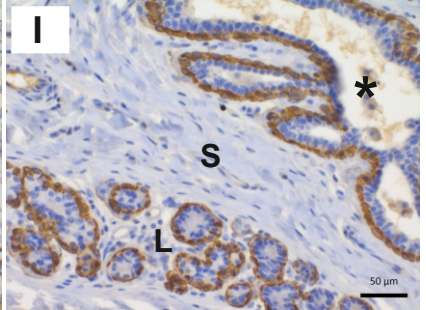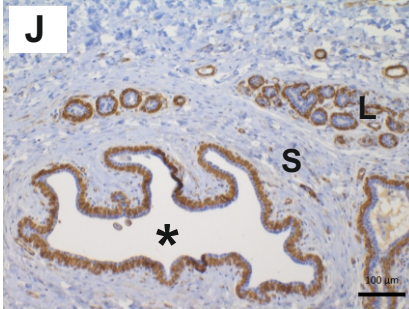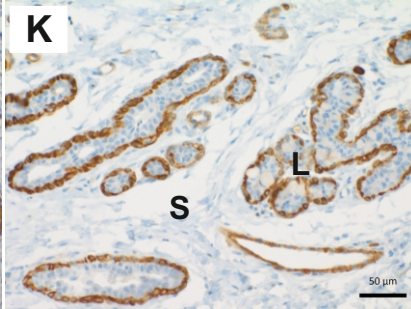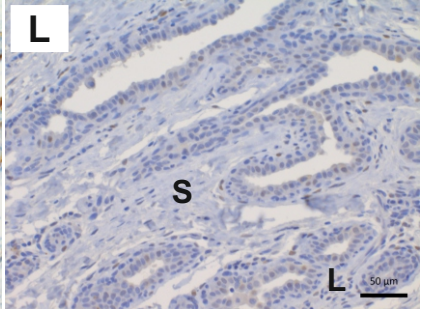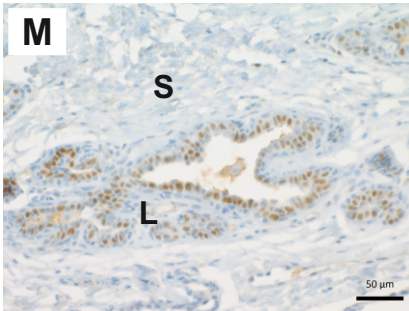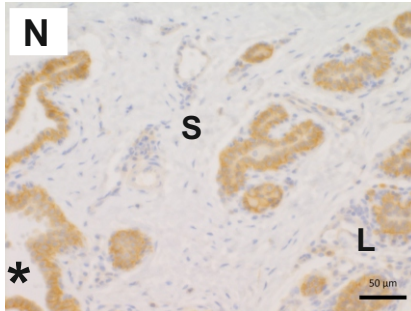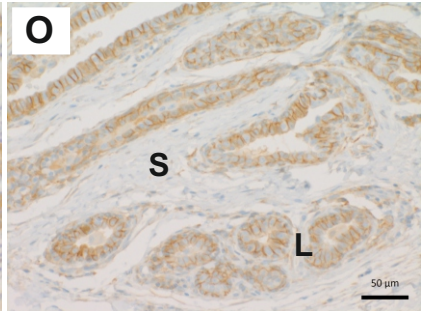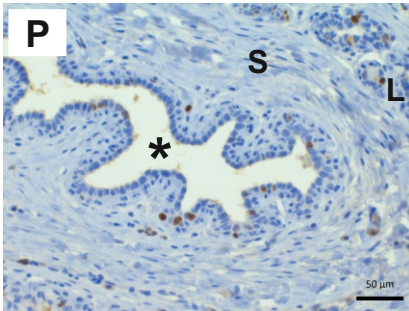

Supplement: Supplementary file 4 — Additional file 4. Figure S3 Histomorphology and biomarker immunohistochemistry of normal canine mammary gland. Structures have been annotated as follows: S=stroma, L=lobule, asterix=duct. A Normal ductolobular morphology. HE. Scale bar 100 μm. B Ductolobular units red, collagenous basement membrane and fibrovascular stroma blue. Masson’s trichrome. Scale bar 100 μm. C High epithelial E-Cadherin expression. Scale bar 50 μm. Counterstain Harris hematoxylin. D High luminal epithelial CK8 expression. Scale bar 50 μm. Counterstain Harris hematoxylin. E Weak-to-moderate luminal epithelial CK19 expression. Scale bar 50 μm. Counterstain Harris hematoxylin. F High luminal epithelial CK18 expression. Scale bar 50 μm. Counterstain Harris hematoxylin. G High basal/ myoepithelial nuclear p63 expression. Scale bar 50 μm. Counterstain Harris hematoxylin. H High basal / myoepithelial CK5 expression. Scale bar 100 μm. Counterstain Harris hematoxylin. I High basal / myoepithelial CK14 expression. Scale bar 50 μm. Counterstain Harris hematoxylin. J High basal / myoepithelial and vascular α-SMA expression. Occasional stromal myofibroblasts are positive. Scale bar 100 μm. Counterstain Harris hematoxylin. K High basal / myoepithelial Calponin expression. Occasional stromal myofibroblasts are positive. Scale bar 50 μm. Counterstain Harris hematoxylin. L Weak-to-moderate nuclear epithelial ERα expression. Scale bar 50 μm. Counterstain Harris hematoxylin. M Moderate-to-high nuclear epithelial PR expression. Scale bar 50 μm. Counterstain Harris hematoxylin. N High membranous (and cytoplasmic) HER2 expression adjacent to investigated tumor mass. Scale bar 50 μm. Counterstain Harris hematoxylin. O Weak-to-moderate membranous EGFR expression. Scale bar 50 μm. Counterstain Harris hematoxylin. P Occasional nuclear epithelial Ki-67 expression. Scale bar 50 μm. Counterstain Harris hematoxylin. [file 12885_2024_13239_MOESM4_ESM.pdf]
